# Effect of a Russian-backbone live-attenuated influenza vaccine with an updated pandemic H1N1 strain on shedding and immunogenicity among children in The Gambia: an open-label, observational, phase 4 study

**DOI:** 10.1016/S2213-2600(19)30086-4

**Published:** 2019-08

**Authors:** Benjamin B Lindsey, Ya Jankey Jagne, Edwin P Armitage, Anika Singanayagam, Hadijatou J Sallah, Sainabou Drammeh, Elina Senghore, Nuredin I Mohammed, David Jeffries, Katja Höschler, John S Tregoning, Adam Meijer, Ed Clarke, Tao Dong, Wendy Barclay, Beate Kampmann, Thushan I de Silva

**Affiliations:** a Vaccines and Immunity Theme, Medical Research Council Unit The Gambia at the London School of Hygiene & Tropical Medicine, Banjul, The Gambia; b Department of Medicine, Imperial College London, London, UK; c Virus Reference Department, Reference Microbiology Services, Public Health England, London, UK; d Centre for Infectious Disease Research, Diagnostics and Laboratory Surveillance, National Institute for Public Health and the Environment, Bilthoven, Netherlands; e Medical Research Council Human Immunology Unit, Weatherall Institute of Molecular Medicine, and Chinese Academy of Medical Science—Oxford Institute, Nuffield Department of Medicine, Oxford University, Oxford, UK; f The Vaccine Centre, London School of Hygiene & Tropical Medicine, Faculty of Infectious and Tropical Diseases, London, UK; g The Florey Institute for Host–Pathogen Interactions and Department of Infection, Immunity and Cardiovascular Disease, University of Sheffield, Sheffield, UK

## Abstract

**Background:**

The efficacy and effectiveness of the pandemic H1N1 (pH1N1) component in live attenuated influenza vaccine (LAIV) is poor. The reasons for this paucity are unclear but could be due to impaired replicative fitness of pH1N1 A/California/07/2009-like (Cal09) strains. We assessed whether an updated pH1N1 strain in the Russian-backbone trivalent LAIV resulted in greater shedding and immunogenicity compared with LAIV with Cal09.

**Methods:**

We did an open-label, prospective, observational, phase 4 study in Sukuta, a periurban area in The Gambia. We enrolled children aged 24–59 months who were clinically well. Children received one dose of the WHO prequalified Russian-backbone trivalent LAIV containing either A/17/California/2009/38 (Cal09) or A/17/New York/15/5364 (NY15) based on their year of enrolment. Primary outcomes were the percentage of children with LAIV strain shedding at day 2 and day 7, haemagglutinin inhibition seroconversion, and an increase in influenza haemagglutinin-specific IgA and T-cell responses at day 21 after LAIV. This study is nested within a randomised controlled trial investigating LAIV–microbiome interactions (NCT02972957).

**Findings:**

Between Feb 8, 2017, and April 12, 2017, 118 children were enrolled and received one dose of the Cal09 LAIV from 2016–17. Between Jan 15, 2018, and March 28, 2018, a separate cohort of 135 children were enrolled and received one dose of the NY15 LAIV from 2017–18, of whom 126 children completed the study. Cal09 showed impaired pH1N1 nasopharyngeal shedding (16 of 118 children [14%, 95% CI 8·0–21·1] with shedding at day 2 after administration of LAIV) compared with H3N2 (54 of 118 [46%, 36·6–55·2]; p<0·0001) and influenza B (95 of 118 [81%, 72·2–87·2]; p<0·0001), along with suboptimal serum antibody (seroconversion in six of 118 [5%, 1·9–10·7]) and T-cell responses (CD4+ interferon γ-positive and/or CD4+ interleukin 2-positive responses in 45 of 111 [41%, 31·3–50·3]). After the switch to NY15, a significant increase in pH1N1 shedding was seen (80 of 126 children [63%, 95% CI 54·4–71·9]; p<0·0001 compared with Cal09), along with improvements in seroconversion (24 of 126 [19%, 13·2–26·8]; p=0·011) and influenza-specific CD4+ T-cell responses (73 of 111 [66%, 60·0–75·6; p=0·00028]). The improvement in pH1N1 seroconversion with NY15 was even greater in children who were seronegative at baseline (24 of 64 children [38%, 95% CI 26·7–49·8] *vs* six of 79 children with Cal09 [8%, 2·8–15·8]; p<0·0001). Persistent shedding to day 7 was independently associated with both seroconversion (odds ratio 12·69, 95% CI 4·1–43·6; p<0·0001) and CD4+ T-cell responses (odds ratio 7·83, 95% CI 2·99–23·5; p<0·0001) by multivariable logistic regression.

**Interpretation:**

The pH1N1 component switch that took place between 2016 and 2018 might have overcome the poor efficacy and effectiveness reported with previous LAIV formulations. LAIV effectiveness against pH1N1 should, therefore, improve in upcoming influenza seasons. Our data highlight the importance of assessing replicative fitness, in addition to antigenicity, when selecting annual LAIV components.

**Funding:**

The Wellcome Trust.

## Introduction

Live attenuated influenza vaccine (LAIV) has been highly efficacious against prepandemic seasonal H1N1 viruses in children, with a meta-analysis^1^ estimating a pooled efficacy of 85% from several randomised controlled trials. However, concerns have been expressed about protection against pandemic H1N1 (pH1N1) influenza using LAIV. Since 2009, when pH1N1 viruses have circulated as the main seasonal H1N1 strain, vaccine effectiveness of the Ann Arbor-backbone LAIV against pH1N1 in the USA has been low, ranging from −21% in 2015–16 to 17% in 2013–14.^2^ This reduction in effectiveness resulted in the Advisory Committee on Immunisation Practices removing their recommendation for LAIV use in 2016.^3^

**Research in context**

**Evidence before this study**

We searched Embase, MEDLINE, Global health, and Web of Science databases up to May 9, 2018, with the terms: (“influenza” OR “flu”) AND (“vaccin*” OR “immuni#ation” OR “Influenza Vaccines [Subject heading]”) AND (“effic*” OR “effect*” OR “immune*” OR “respons*” OR “protect*”) AND (“Africa” OR “Africa [Subject heading]” OR each African country [defined by the UN]). This search strategy identified no live attenuated influenza vaccine (LAIV) immunogenicity studies and only two LAIV efficacy studies in African children. The first study was of the Ann-Arbor LAIV in 2001–02, when pre-pandemic H1N1 was circulating. This study was a multicentre randomised placebo-controlled trial that included 277 children aged 6–36 months from South Africa. The efficacy of LAIV in this subset of children was 87% (95% CI 64–95). The second study was a single-centre, randomised, placebo-controlled trial of Russian-backbone LAIV in children aged 2–5 years in Senegal. The study was done in 2013 and, therefore, included pandemic H1N1 (pH1N1). The efficacy against vaccine-matched isolates was −6·1% (95% CI −50·0 to 25·0), and pH1N1 was the predominant circulating strain. In the 2017–18 season, the pH1N1 component in LAIV was updated for the first time in both the Ann Arbor and Russian-backbone LAIVs, with A/Michigan/45/2015-like strains. No studies have been published about whether this change has affected the shedding and immunogenicity of pH1N1 in LAIV.

**Added value of this study**

Our findings show that A/17/California/2009/38 pH1N1 strain shedding and immunogenicity is less than that of H3N2 and influenza B in the Russian-backbone LAIV, providing an explanation for the lack of efficacy seen in the randomised controlled trial in Senegal. Our data suggest this observation is not attributable to reduced pH1N1 vaccine take secondary to pre-existing immune responses. Our findings show for the first time that updating the Russian-backbone LAIV pH1N1 component has resulted in a vaccine with significantly greater nasopharyngeal shedding, seroconversion, and influenza-specific T-cell induction to pH1N1. We are able to model this difference in replicative ability of old and new pH1N1 LAIV components in vitro.

**Implications of all the available evidence**

Impaired replicative ability of pH1N1 components in LAIV might have caused recent low efficacy and effectiveness of LAIV. An improvement in protection against pH1N1 can be expected in the future. These data highlight the importance of assessing viral replicative fitness in addition to antigenicity when selecting vaccine formulations. Studies are needed to ascertain whether improved shedding and immunogenicity translates into improved efficacy and effectiveness. Further research is needed to understand the genetic factors that underlie these phenotypes in vaccine strains to design more rational choices of vaccine antigens for LAIV.

A randomised controlled trial of Russian-backbone LAIV (Nasovac-S; Serum Institute of India Pvt, Pune, India) among children aged 2–5 years in Senegal did not show efficacy (0·0%, 95% CI −26·4 to 20·9) in 2013, when pH1N1 was the predominant circulating vaccine-matched virus.^4^ Both LAIV formulations in these studies contained haemagglutinin and neuraminidase from pH1N1 A/California/07/2009-like (Cal09) viruses. It is unclear why protection conferred by the pH1N1 component in LAIV has been suboptimal. Potential reasons include pre-existing immunity, poor viral replicative fitness, or competition from other co-formulated strains—all limiting pH1N1 take and immunogenicity.^3^

The Russian-backbone LAIV (Nasovac-S) was granted a WHO prequalification certificate in 2015, opening up the potential for use in low-income and middle-income countries. The findings in Senegal with this vaccine are especially pertinent because the burden of influenza in Africa is high; influenza-related admissions to hospital in children younger than 5 years are approximately threefold higher than in Europe.^5^ Superior efficacy of LAIV over inactivated influenza vaccine in young children (predominantly in high-income settings),^1^ needle-free delivery, and lower manufacturing costs make LAIV an attractive option to tackle this burden in Africa.^3^ However, few LAIV studies have been done in African cohorts and no published immunogenicity data are available from African children to date.^6^ In particular, the absence of immunological endpoints from the randomised controlled trial in Senegal makes it difficult to understand the reasons for the lack of efficacy recorded.^4^

In 2017–18, the pH1N1 Cal09 strain (A/17/California/2009/38) was updated according to WHO recommendations to an A/Michigan/45/2015-like strain (A/17/New York/15/5364 [NY15]), following antigenic drift. This first-ever recommended update to pH1N1 provided a unique opportunity to compare replicative ability and immunogenicity of these two pH1N1 strains. To understand how differences in strain shedding and immunogenicity might account for the findings of the Senegal trial, we compared one cohort of influenza vaccine-naive Gambian children vaccinated with the Russian-backbone Cal09 LAIV formulation from 2016–17 with a second cohort vaccinated with the NY15 LAIV formulation from 2017–18.

## Methods

### Study design and participants

We did an open-label, prospective, observational, phase 4 immunogenicity study in Sukuta, a periurban area in The Gambia. Our study is nested within a larger randomised trial comparing microbiome changes in children assigned LAIV with changes in unvaccinated children (NCT02972957; appendix pp 3, 4). Data in our study are from all children enrolled in the randomised trial who were given LAIV. After community sensitisation, parents expressing an interest in the randomised study were invited for consent discussions. Eligible children had to be aged 24–59 months and clinically well, with no history of respiratory illness within the past 14 days (appendix p 2).

This study was approved by The Gambia Government and UK Medical Research Council (MRC) joint ethics committee and the Medicines Control Agency of The Gambia, and it was done according to International Conference on Harmonisation Good Clinical Practice standards. A parent provided written or thumbprinted informed consent for their child or children to participate. If parents were not English literate, an impartial witness was present throughout the informed consent discussion undertaken in a local language, who signed to confirm completeness of the consent provided.

### Procedures

When LAIV was updated in 2017–18, haemagglutinin and neuraminidase from pH1N1 Cal09 were replaced with those from NY15, whereas identical H3N2 (A/17/Hong Kong/2014/8296) and B/Vic (B/Texas/02/2013 [Victoria lineage]) strains were used. Vaccine titres per dose (50% egg infectious dose equivalents [EID50eq] per mL) were 1 × 10^8·0^ for pH1N1, 1 × 10^7·5^ for H3N2, 1 × 10^7·2^ for B/Vic in the 2016–17 LAIV and 1 × 10^7·7^ for pH1N1, 1 × 10^7·6^ for H3N2, 1 × 10^7·3^ for B/Vic in the 2017–18 LAIV.

The study was done outside the peak influenza transmission season (June to October) based on surveillance data from Senegal and unpublished data from studies in The Gambia.^7^ Children received one dose of intranasal trivalent Russian-backbone LAIV (Nasovac-S; northern hemisphere formulation) in either 2017 (the Cal09 strain from 2016–17) or 2018 (the NY15 strain from 2017–18 formulation). Children received the vaccine formulation that corresponded with their year of enrolment.

Nasopharyngeal swabs were taken before vaccination (day 0), on day 2, and on day 7 using flocked swabs (FLOQSwabs; Copan, Murrieta, CA, USA). We obtained buccal cavity oral fluid with swabs (Oracol Plus; Malvern Medical Development, Worcester, UK) on day 0 and day 21. Whole blood samples were obtained for flow cytometry and serum separation on day 0 and day 21. We chose day 21 to measure vaccine response, in line with previous work.^8^, ^9^, ^10^ Nasopharyngeal swabs, oral fluid, and serum samples were stored at −70°C before further processing.

Haemagglutinin inhibition assays were done according to standard methods,^11^ using vaccine haemagglutinin-matched and neuraminidase-matched viruses. Seroconversion was defined as a fourfold or greater titre increase (to ≥1:40) from day 0 to day 21. Total and influenza haemagglutinin-specific IgA in oral fluid was detected using a previously described ELISA,^12^ using recombinant vaccine-matched haemagglutinin. Samples were assayed at dilutions ranging from 1:1000 to 1:20000 for total IgA and from undiluted to 1:16 for influenza-specific IgA, and samples were quantified using an IgA standard curve. Undiluted samples with influenza-specific IgA below the limit of quantitation (LOQ) were assigned LOQ values. We calculated the fold change in the proportion of influenza-specific IgA to total IgA from day 0 to day 21. A twofold increase was considered a significant response.^13^

T-cell responses were quantified by stimulating fresh whole blood (200 μL) on the day of collection for 18 h with overlapping 15–18-mer peptide pools (2 μg/mL) covering vaccine-matched whole haemagglutinin, matrix and nucleoprotein, and co-stimulatory antibodies (antiCD28 and antiCD49; BD Biosciences, Franklin Lakes, NJ, USA). Influenza B responses were measured in 2018 only. We did intracellular cytokine staining for interferon (IFN)γ and interleukin (IL)2 and analysed cells with a flow cytometer (LSR Fortessa; BD Biosciences; appendix pp 5, 6).^14^ Responses in negative controls (antiCD28 and antiCD49) were subtracted from peptide-stimulated conditions before further analysis; negative values were set to zero. To avoid systematic bias in adjusting for negative values alone, we set a threshold (based on the distribution of negative values; appendix p 7) below which all positive values were also considered a non-response, as described previously.^14^, ^15^ In analyses calculating the fold change from day 0 to day 21, null responses were assigned a value halfway between zero and this threshold. A twofold increase after LAIV was considered a significant response.

Vaccine shedding from nasopharyngeal swabs was assessed with monoplex reverse-transcriptase PCR (RT-PCR) using haemagglutinin-specific primers and probes (appendix p 8). In 2018, fully quantitative RT-PCR results were obtained by inclusion of a standard curve with known vaccine titres (log_10_EID50eq per mL; appendix p 9). RT-PCR assays with primers and probes mapping to internal genes were used to distinguish LAIV from seasonal influenza viruses (appendix p 8).^16^ Despite optimisation of assay conditions, maximum LAIV dilutions detected by LAIV-specific RT-PCR were at least one log_10_ lower than those detected by haemagglutinin-specific RT-PCR (appendix p 10). Therefore, only samples with cycle threshold (ct) values of 30 or lower in seasonal influenza assays were tested with LAIV-specific assays, with 100% confirmed as LAIV strains.

Primary human nasal epithelial cell cultures (MucilAir; Epithelix Sàrl, Geneva, Switzerland) were used for in vitro viral replication experiments. Madin-Darby Canine Kidney (MDCK) cells (ATCC, Manassas, VA, USA) and MDCK-SIAT cells (WHO Collaborating Centre for Reference and Research on Influenza, London, UK) were maintained at 37°C with 5% CO_2_ in Dulbecco's modified Eagle's Medium (DMEM; Gibco-Life Technologies, Waltham, MA, USA) supplemented with 10% fetal bovine serum, 1% penicillin–streptomycin, and 1% non-essential amino acids. We also added 1 mg/mL G418 (Gibco-Life Technologies) for MDCK-SIAT cells. Viral stocks of Nasovac-S monovalent forms were titrated by plaque assay at 32°C on MDCK cells (for pH1N1 and influenza B) or MDCK-SIAT cells (for H3N2). Apical surfaces of human nasal epithelial cells were inoculated with each monovalent virus (multiplicity of infection 0·01 plaque-forming units per cell) for 1 h at 32°C and 5% CO_2_ in triplicate. The inoculum was removed and the apical surface of the human nasal epithelial cells was washed with DMEM before incubation at 32°C. At indicated timepoints (days 1–6 after inoculation), DMEM was added to the human nasal epithelial cells and incubated for 30 min, then it was removed and stored; the stored DMEM—containing virions from the epithelial cell cultures—was titrated by plaque assay. Experiments were done on two separate occasions using cells from different donors.

To assess the acid stability of pH1N1 strains, Cal09 or NY15 were mixed with pH-adjusted MES (2-[N-morpholino]ethanesulphonic acid) buffer (100 mmol/L MES, 150 mol/L sodium chloride, 0·9 mol/L calcium chloride, 0·5 mol/L magnesium chloride) in triplicate (1:10 dilution) and the mixture was incubated for 15 min at room temperature. The buffer was inactivated with DMEM and infectious virus was titrated by plaque assay.

### Outcomes

Primary shedding and immunogenicity outcomes were the percentage of children with LAIV strain shedding at day 2 and day 7, haemagglutinin inhibition seroconversion, and an increase in influenza haemagglutinin-specific IgA and T-cell responses at day 21 after LAIV.

### Statistical analysis

The sample size calculation was based on LAIV microbiome endpoints not presented here (appendix p 3). Differences in unpaired proportions (shedding, seroconversion, IgA responses, and T-cell responses) between years (2017 and 2018) were assessed with either the χ^2^ test or Fisher's exact test. Differences in continuous variables (ct value, log_10_EID50eq per mL, and geometric mean fold change in haemagglutinin inhibition) between years (2017 and 2018) or serostatus (positive or negative) were assessed with either the unpaired *t* test or Mann-Whitney *U* test. Differences in viral load (log_10_EID50eq per mL) between strains (NY15, H3N2, and influenza B) within the same visit (paired data) were assessed with the Friedman test (with Dunn's post-test for multiple comparisons). Pairwise viral load correlations were assessed using Spearman's rank-order correlation (r_s_). Correlation coefficients were interpreted as low (r_s_=0·30–0·49), moderate (r_s_=0·50–0·69), high (r_s_=0·70–0·89), or very high (r_s_=0·90–1·00). The Wilcoxon signed-rank test was used to compare T-cell responses before and after vaccination. Separate logistic regression analyses were done for dependent variables (shedding, seroconversion, T-cell responses, and IgA responses). Independent variables were selected for multivariable logistic regression models if biologically relevant and the p value from univariable regression was less than 0·2. Each multivariable logistic regression model always included year, age, and sex as potential confounders. Viral loads were ascertained from standard curves using Python version 3.6 (SciPy package). In vitro viral replication was quantified using the area under the curve function in GraphPad Prism 5.0d (GraphPad Software, San Diego, CA, USA) and compared between NY15 and Cal09 strains using an unpaired *t* test. The proportion of monofunctional and dual-functional T-cell responses were estimated using Boolean gating on FlowJo 10.4 (FlowJo LLC, Ashland, OR, USA) and statistical significance between timepoints tested with the Permutation test in SPICE (version 6.0).^15^ Proportions are displayed with 95% CIs. All tests were two-sided at the 5% significance level and were Bonferroni-adjusted for multiple comparisons within each set of analyses. Statistical analyses were done using R version 3.5.1, Stata release 12 (StataCorp, College Station, TX, USA), and GraphPad Prism 5.0d.

### Role of the funding source

The funder had no role in study design, data collection, data analysis, data interpretation, or writing of the report. BBL, TIdS, EPA, YJJ, NIM, DJ, KH, and AS had access to raw data. The corresponding author had full access to all data in the study and had final responsibility for the decision to submit for publication.

## Results

**Figure 1:**
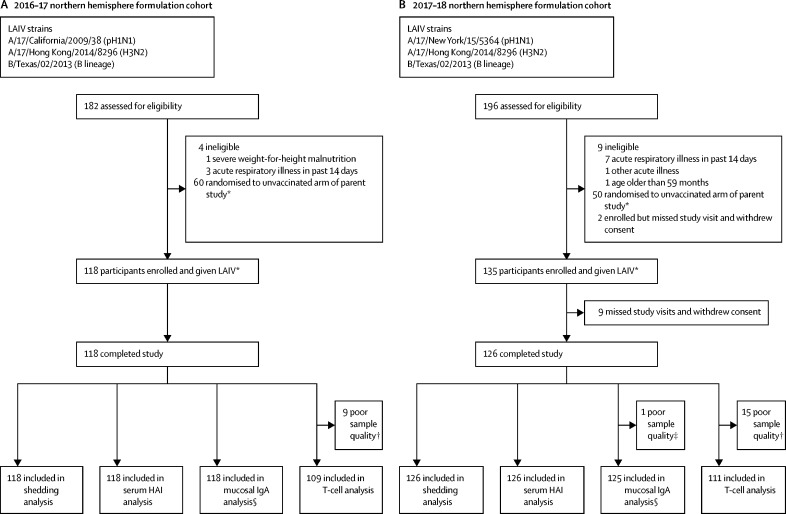
Study profile Overview of participants who received (A) the 2016–17 northern hemisphere Russian-backbone LAIV formulation and (B) the 2017–18 northern hemisphere Russian-backbone LAIV formulation. LAIV=live attenuated influenza vaccine. pH1N1=pandemic H1N1. HAI=haemagglutinin inhibition. *The study was nested within a larger randomised controlled trial (NCT02972957; appendix pp 3,4). †Sparse cell populations seen on flow cytometry. ‡Total IgA not detected in sample. §No pH1N1 data for one sample in 2016–17 cohort and no pH1N1 data for four samples and H3N2 data for three samples in 2017–18 cohort because of inadequate sample volume.

Between Feb 8, 2017, and April 12, 2017, 118 children were enrolled and received one dose of the 2016–17 northern hemisphere formulation LAIV (Cal09 pH1N1; figure 1A). Between Jan 15, 2018, and March 28, 2018, a separate cohort of 135 children were enrolled and received one dose of the 2017–18 northern hemisphere formulation LAIV (NY15 pH1N1; figure 1B). 118 children in 2017 and 126 children in 2018 completed the study. All study visits were within protocol-defined windows (+1 day for day 2 visit, +7 days for day 7 visits, and +7 days for day 21 visits). In the 2017 cohort, 118 (100%) of 118 day 2 visits were 2 days after LAIV, 115 (97%) of 118 day 7 visits were 7 days after LAIV (three visits were 8 days, 12 days, and 14 days after LAIV), and 112 (95%) of 118 day 21 visits were 21 days after LAIV (five visits were 22 days after LAIV and one was 25 days after LAIV). In the 2018 cohort, 122 (97%) of 126 day 2 visits were 2 days after LAIV (four visits were 3 days after LAIV), 119 (94%) of 126 day 7 visits were 7 days after LAIV (seven visits were 8 days after LAIV), and 117 (93%) of 126 day 21 visits were 21 days after LAIV (eight visits were 22 days after LAIV and one visit was 26 days after LAIV). Baseline demographics did not differ significantly between the two cohorts with the exception of baseline haemagglutinin inhibition titres (table).

**Table:** Demographic characteristics and baseline influenza serological data

		**2016–17 LAIV (n=118)**	**2017–18 LAIV (n=126)**	**p value**
Age (months)		35·1 (28·3–44·9)	35·3 (28·0–40·5)	0·44
Sex		··	··	0·61
	Female	57 (48%)	56 (44%)	··
	Male	61 (52%)	70 (56%)	··
Height (cm)		92·9 (7·4)	91·8 (6·3)	0·23
Weight (kg)		12·9 (2·1)	12·6 (1·7)	0·30
Weight-for-height malnutrition*		··	··	0·93
	None	76 (64%)	82 (65%)	··
	Mild	33 (28%)	36 (29%)	··
	Moderate	9 (8%)	8 (6%)	··
Tribe		··	··	0·27
	Mandinka	96 (81%)	99 (79%)	··
	Wolof	5 (4%)	7 (6%)	··
	Fula	3 (3%)	5 (4%)	··
	Jola	6 (5%)	4 (3%)	··
	Serehule	2 (2%)	5 (4%)	··
	Serere	5 (4%)	1 (1%)	··
	Other	1 (1%)	5 (4%)	··
History of ever being admitted to hospital with a respiratory infection		6 (5%)	3 (2%)	0·32
History of more than one respiratory infection needing medication in the past year		8 (7%)	13 (10%)	0·37
Age when stopped breastfeeding (months)		20 (18–24)	20 (18–24)†	0·80
Baseline seropositive (haemagglutinin inhibition titre ≥1:10)				
	pH1N1‡	39 (33%)	62 (49%)	0·013
	H3N2	90 (76%)	70 (56%)	0·00070
	B/Vic	25 (21%)	54 (43%)	0·00040
Haemagglutinin inhibition titre in children seropositive at baseline				
	pH1N1‡	160 (80–160)	226 (160–320)	0·00050
	H3N2	160 (80–160)	160 (80–320)	0·16
	B/Vic	160 (80–226·3)	226 (160–320)	0·015

Data are n (%), median (IQR), or mean (SD). pH1N1=pandemic H1N1.

* Malnutrition was categorised based on weight-for-height SD (*Z* score): none (>–1), mild (–2 to <–1), moderate (–3 to <–2). Children with severe malnutrition (weight-for-height SD <–3) were excluded.

† Missing data for two children.

‡ pH1N1 virus used for serum haemagglutinin inhibition assays was changed for the cohort given 2017–18 LAIV to reflect the update from Cal09 to NY15.

**Figure 2:**
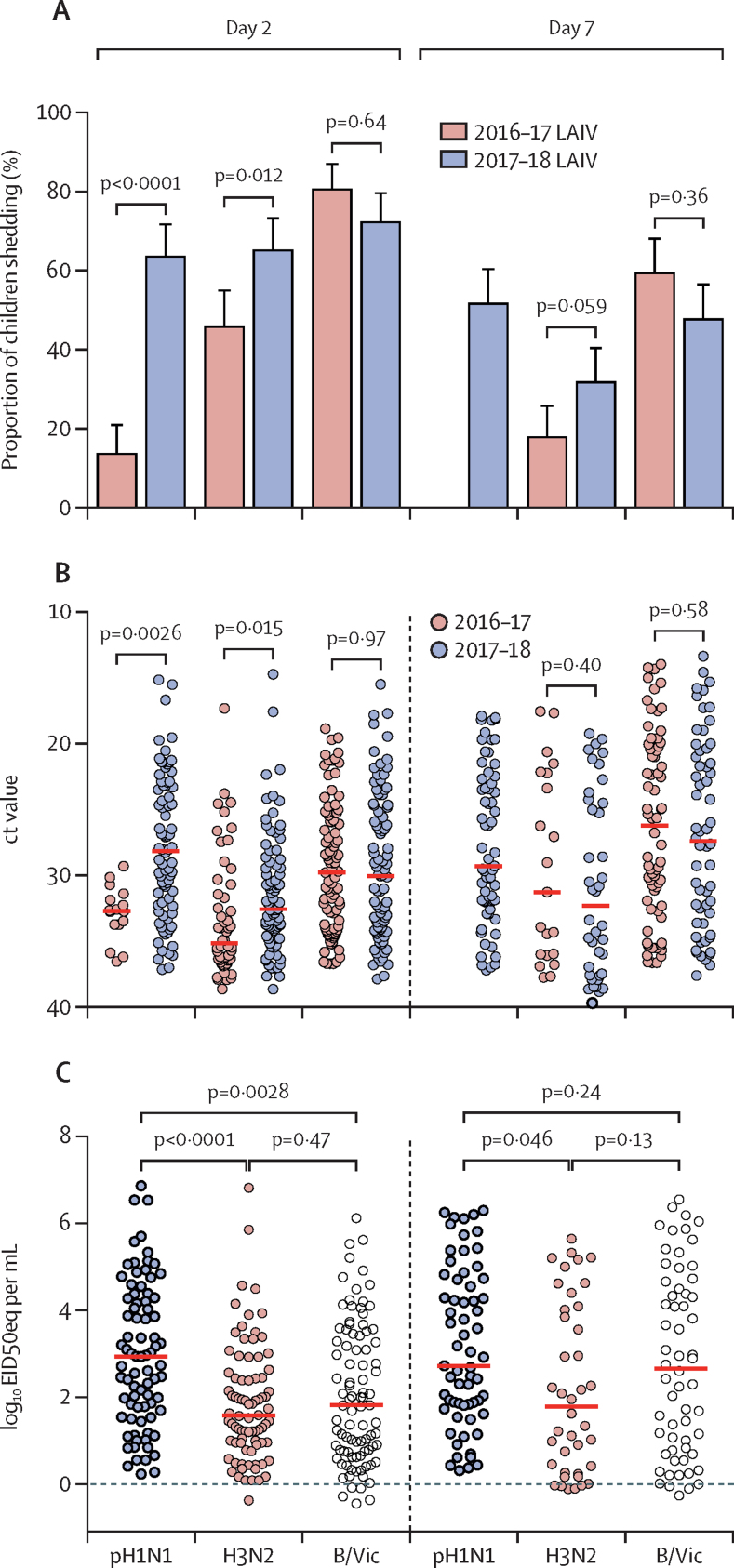
Shedding of strains in the nasopharynx after vaccination (A) Percentage of children shedding vaccine virus with 2016–17 LAIV formulation compared with the 2017–18 LAIV formulation, at day 2 and day 7. Error bars represent the upper 95% CI. (B) Viral load in the nasopharynx is indicated by ct values from RT-PCR. Red bars indicate median ct values. Lower ct values indicate higher viral loads. (C) Quantitative RT-PCR viral load in children from the 2018 cohort for each strain. Red bars indicate median values. p values are Bonferroni-adjusted for multiplicity within each group of analyses. LAIV=live attenuated influenza vaccine. pH1N1=pandemic H1N1. H3N2=A/17/Hong Kong/2014/8296. B/Vic=B/Texas/02/2013 (Victoria lineage). ct=cycle threshold. RT-PCR=reverse-transcriptase PCR. EID50eq=50% egg infectious dose equivalents.

No influenza strains were detected from nasopharyngeal swabs taken immediately before vaccination in any children. After administration of the 2016–17 LAIV, pH1N1 Cal09 shedding was seen in significantly fewer children (16 of 118 [14%, 95% CI 8·0–21·1]) at day 2 compared with H3N2 (54 of 118 [46%, 36·6–55·2]; p<0·0001) and B/Vic (95 of 118 [81%, 72·2–87·2]; p<0·0001; figure 2). No pH1N1 Cal09 shedding was recorded at day 7 with the 2016–17 LAIV, with H3N2 shedding noted in 21 of 118 children (18%, 95% CI 11·4–25·9) and B/Vic shedding in 70 of 118 children (59%, 49·9–68·3). Administration of the 2017–18 LAIV resulted in significantly more children shedding pH1N1 NY15 at day 2 (80 of 126 [63%, 95% CI 54·4–71·9]; p<0·0001 compared with pH1N1 Cal09; figure 2A), with shedding of H3N2 seen in 82 of 126 children (65%, 95% CI 56·1–73·4) and shedding of B/Vic reported in 91 of 126 children (72%, 63·5–79·8). Shedding of pH1N1 NY15 was detected at day 7 with the 2017–18 LAIV (65 of 126 children [52%, 42·5–60·6]), with shedding of H3N2 recorded in 40 of 126 children (32%, 95% CI 23·7–40·6) and shedding of B/Vic noted in 60 of 126 children (48%, 38·7–56·7).

Significantly higher pH1N1 nasopharyngeal viral loads (lower ct values) were also seen with the 2017–18 LAIV compared with the 2016–17 LAIV at day 2 (p=0·0026; figure 2B). Quantitative RT-PCR data showed that pH1N1 NY15 viral loads from the 2017–18 LAIV were significantly higher than H3N2 (p<0·0001) and B/Vic (p=0·0028) at day 2 (figure 2C). To investigate whether the improved replication of pH1N1 with the 2017–18 LAIV resulted in greater competition with H3N2 and B/Vic and, therefore, lower viral loads of these strains, viral loads were compared in co-shedders of each strain. No significant negative effect on H3N2 and B/Vic replication was reported, with a significant positive correlation between pH1N1 and H3N2 shedding noted at day 2 (low correlation, r_s_=0·40; p=0·0012) and day 7 (moderate correlation, r_s_=0·51; p=0·0032; appendix p 11).

**Figure 3:**
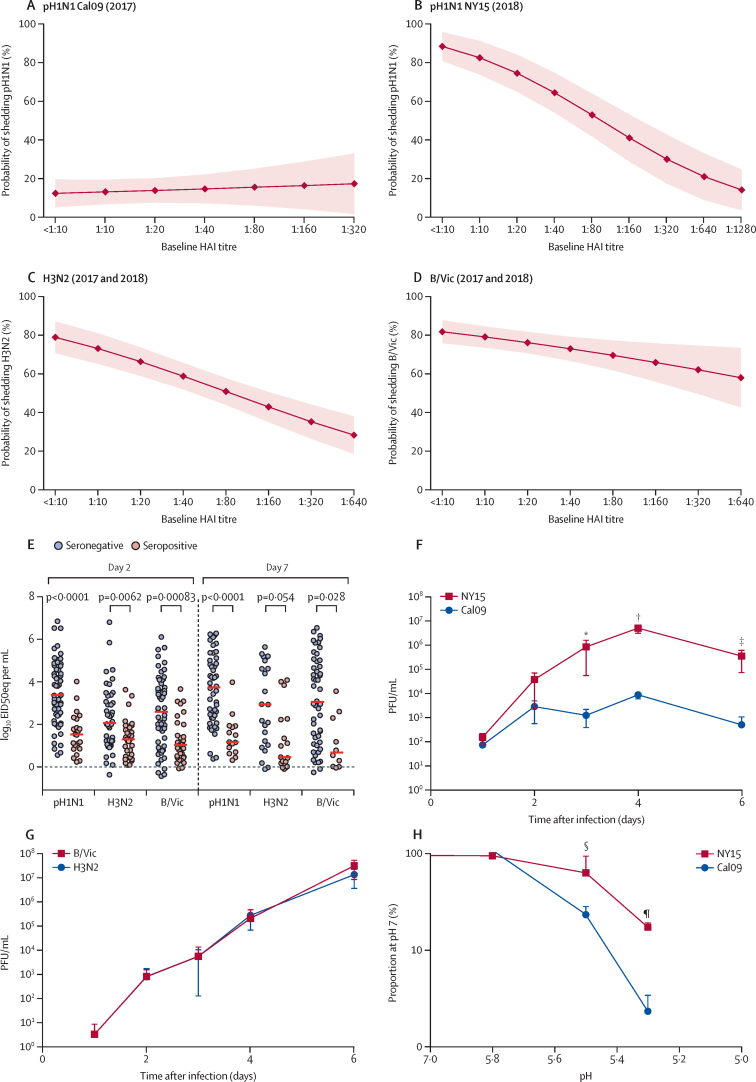
Effect of baseline serum antibody on LAIV strain shedding in the nasopharynx and replicative ability of viruses in primary epithelial cell cultures (A–D) Predicted probability from logistic regression of vaccine strain shedding at day 2 after LAIV at a given baseline serum HAI titre to each matched strain. Dots show predicted proportions and shaded areas show 95% CIs. Data shown for Cal09 pH1N1 (A), NY15 pH1N1 (B), H3N2 (C), and B/Vic (D). Upper limit is based on maximum observed HAI titre in the dataset. When data from 2017 and 2018 were combined for H3N2 and B/Vic, results were adjusted for year (appendix p 12). (E) Nasopharyngeal viral load at day 2 and day 7 after 2017–18 LAIV, with participants stratified by baseline serostatus to vaccine haemagglutinin-matched and neuraminidase-matched influenza strains. Red bars indicate median values. (F and G) Replication of pH1N1 (F) or H3N2 and B/Vic (G) vaccine strains in primary nasal epithelium. Dots denote mean values and errors bars the SD. In (F), p<0·0001 comparing area under the curve. (H) Effect of pH on vaccine strain growth in vitro. Dots denote mean values and errors bars the SD. The y axis is a logarithmic scale. LAIV=live attenuated influenza vaccine. HAI=haemagglutinin inhibition. pH1N1=pandemic H1N1. Cal09=A/17/California/2009/38. NY15=A/17/New York/15/5364. H3N2=A/17/Hong Kong/2014/8296. B/Vic=B/Texas/02/2013 (Victoria lineage). EID50eq=50% egg infectious dose equivalents. PFU=plaque-forming units. p values for specific timepoints are *p=0·047, †p=0·0019, ‡p=0·029, §p=0·013, and ¶p<0·0001.

To ascertain whether pre-existing adaptive immunity accounted for poor pH1N1 Cal09 shedding, univariable logistic regression was done to calculate the predicted probability of shedding at each baseline haemagglutinin inhibition titre (figure 3), adjusting for year in the H3N2 and B/Vic models. Although an inverse relation was evident for H3N2 (figure 3C) and B/Vic (figure 3D) between baseline haemagglutinin inhibition titre and shedding, this relation was not evident for Cal09 (figure 3A), for which low shedding was predicted even in seronegative children. By contrast, NY15 shedding was inversely related to the magnitude of the baseline haemagglutinin inhibition titre (figure 3B). Logistic regression also showed no associations between shedding and prevaccination T-cell responses or haemagglutinin-specific mucosal IgA responses for Cal09 (appendix p 12). Similarly, no association was seen between T-cell responses or IgA responses and shedding for H3N2 or B/Vic strains, after adjusting for baseline haemagglutinin inhibition titre (appendix p 12). Significantly lower nasopharyngeal viral loads were recorded at day 2 and day 7 for all three strains in baseline seropositive children compared with baseline seronegative children who received the 2017–18 LAIV formulation (figure 3E), further emphasising the importance of serum antibody in this process.

In the seronegative population, shedding of pH1N1 Cal09 at day 2 was reported in ten of 79 children (13%, 95% CI 7·0–21·8) whereas shedding of pH1N1 NY15 was noted in 58 of 64 children (91%, 81·0–95·6; p<0·0001). Shedding of H3N2 with the 2016–17 LAIV was seen in 21 of 28 seronegative children (75·0%, 95% CI 56·6–87·3) and with the 2017–18 LAIV, shedding of H3N2 was recorded in 46 of 56 seronegative children (82%, 70·2–90·0; p=0·63). In the same seronegative population, shedding of B/Vic with the 2016–17 LAIV was detected in 78 of 93 children (84%, 95% CI 75·1–90·0) and, with the 2017–18 LAIV, shedding of B/Vic was recorded in 57 of 72 children (79%, 68·4–86·9; p=0·57). Comparisons of ct values between 2016–17 and 2017–18 LAIV strains in seronegative children showed a lower ct value (higher viral load) at day 2 with pH1N1 NY15 compared with pH1N1 Cal09 (p<0·0001; appendix p 13).

Monovalent vaccine strain replication was tested in primary human nasal epithelial cells cultured at an air–liquid interface to see whether in-vitro kinetics (in the absence of adaptive immune responses) reflected Cal09 and NY15 pH1N1 shedding in children. NY15 replication was greater than Cal09 replication (figure 3F), whereas H3N2 and B/Vic growth was equivalent (figure 3G). Since stability in acidic environments in the upper respiratory tract could be important for replicative ability, Cal09 and NY15 were quantified after exposure to varying pH levels. Greater stability of NY15 was seen in acidic environments compared with Cal09 (figure 3H).

**Figure 4:**
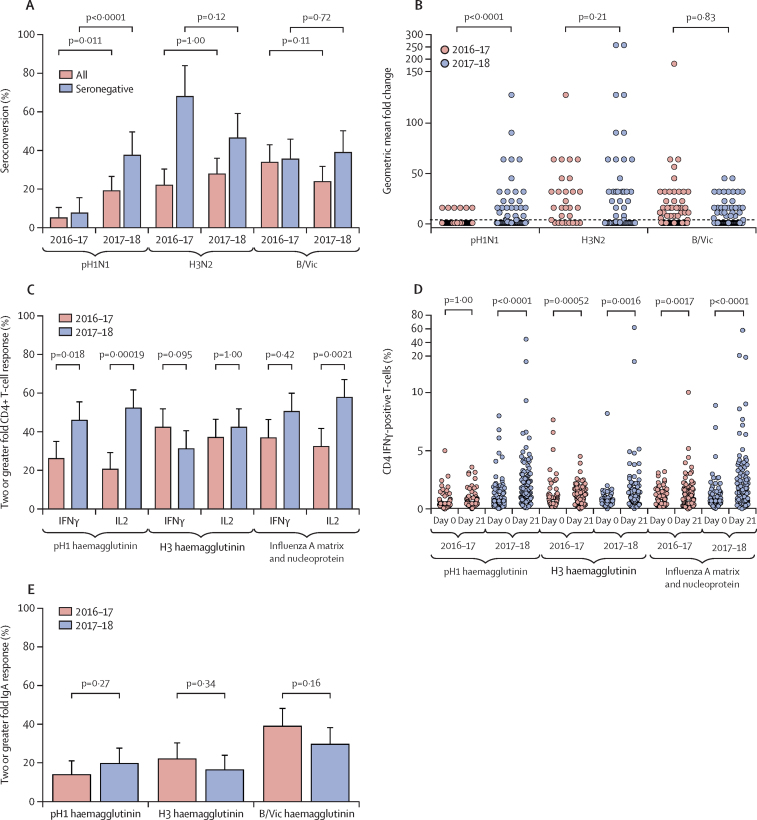
Immunogenicity to pH1N1 with the 2016–17 and 2017–18 LAIV formulations p values are Bonferroni-adjusted for multiplicity within each group of analyses. (A) Percentage of children seroconverting to each LAIV strain, comparing 2016–17 and 2017–18 formulations. Error bars represent the upper 95% CI. (B) Geometric mean fold change in serum haemagglutinin inhibition titre from baseline to day 21, comparing children seronegative at baseline given 2016–17 and 2017–18 LAIVs. Dotted line depicts a fold change of four. y axis is a logarithmic scale. (C) Influenza-specific CD4+ T-cell responses to vaccine strain-matched pH1 haemagglutinin (Cal09 in 2016–17 or NY15 in 2017–18), H3 haemagglutinin, influenza A matrix and nucleoprotein (both matched to LAIV backbone) peptide pools, comparing 2016–17 and 2017–18 LAIVs. Error bars represent the upper 95% CI. (D) Percentage of children with a twofold rise in influenza-specific CD4+ T-cell responses at day 21 after 2016–17 and 2017–18 LAIVs. y axis is a logarithmic scale. (E) Percentage of influenza-specific mucosal IgA responders given the 2016–17 and 2017–18 LAIVs. Error bars represent the upper 95% CI. pH1N1=pandemic H1N1. LAIV=live attenuated influenza vaccine. Cal09=A/17/California/2009/38. NY15=A/17/New York/15/5364. H3N2=A/17/Hong Kong/2014/8296. B/Vic=B/Texas/02/2013 (Victoria lineage). IFNγ=interferon γ.

The 2016–17 LAIV resulted in significantly fewer children seroconverting to pH1N1 Cal09 (six of 118 [5%, 95% CI 1·9–10·7]) compared with H3N2 (26 of 118 [22%, 14·9–30·6]; p=0·00030) and B/Vic (40 of 118 [34%, 25·4–43·2]; p<0·0001). A significant increase was recorded in pH1N1 NY15 seroconversion with the 2017–18 LAIV compared with Cal09 (24 of 126 [19%, 95% CI 13·2–26·8]; p=0·011; figure 4A), with no difference in H3N2 (35 of 126 [28%, 20·7–36·2]; p=1·00) or B/Vic (30 of 126 [24%, 17·2–32·0]; p=0·11). The improved seroconversion to pH1N1 with NY15 compared with Cal09 was especially evident in seronegative children (24 of 64 [38%, 95% CI 26·7–49·8] *vs* six of 79 [8%, 2·8–15·8]; p<0·0001; figure 4A), with a significant difference in geometric mean fold change in haemagglutinin inhibition for pH1N1 NY15 in 2017–18 compared with pH1N1 Cal09 in 2016–17 (p<0·0001; figure 4B).

Influenza-specific CD4+ IFNγ-positive, CD4+ IL2-positive, and CD8+ IFNγ-positive T-cell responses were detected at baseline and after vaccination. Although the magnitude of CD8+ responses was generally higher, LAIV-induced responses were predominantly CD4+ (figures 4C and 4D; appendix pp 14, 15). The 2016–17 LAIV did not induce significant pH1 haemagglutinin-specific CD4+ IFNγ-positive or CD4+ IL2-positive responses, whereas H3 haemagglutinin-positive and influenza A matrix and nucleoprotein-specific responses were significantly increased from baseline (figure 4D; appendix p 14). By contrast, the 2017–18 LAIV induced significant pH1 haemagglutinin-specific CD4+ T-cells at day 21. Accordingly, a twofold or greater rise in pH1 haemagglutinin-specific CD4+ T-cell responses was noted in more children given the 2017–18 LAIV than in those given the 2016–17 LAIV (50 of 109 [46%, 95% CI 36·3–55·7] *vs* 29 of 111 [26%, 18·2–35·3] for CD4+ IFNγ-positive responses; and 57 of 109 [52%, 42·5–61·9] *vs* 23 of 111 [20·7%, 13·6–29·5] for CD4+ IL2-positive responses; figure 4C). A twofold or greater rise in CD4+ IFNγ-positive and/or CD4+ IL2-positive responses was recorded in 45 of 111 children (41%, 95% CI 31·3–50·3) given the 2016–17 LAIV and in 73 of 111 children (66%, 60·0–75·6) given the 2017–18 LAIV. B/Vic haemagglutinin-specific and influenza B matrix and nucleoprotein-specific CD4+ responses were also induced (appendix p 15). No significant change in the proportion of monofunctional or dual-functional CD4+ T-cell responses was seen after vaccination (appendix p 15). Influenza-specific mucosal IgA responses to pH1N1 did not differ between the 2016–17 LAIV (16 of 117 children [14%, 95% CI 8·0–21·3]) and the 2017–18 LAIV (24 of 121 children [20%, 13·1–28·1]; p=0·27; figure 4E).

The effect of shedding on immunogenicity was investigated using H3N2 data (the largest sample of participants immunised with the same antigen and with T-cell data available). Seroconversion and T-cell responses were highest in children with shedding at both days 2 and 7 (appendix pp 16, 17). Multivariable logistic regression showed a significant effect of this prolonged shedding on the odds of seroconversion (odds ratio [OR] 12·69, 95% CI 4·1–43·6; p<0·0001) and CD4+ T-cell responses (7·83, 2·99–23·5; p<0·0001; appendix pp 16, 17). No such relation was seen with IgA responses (appendix pp 16–18). The odds of seroconversion were also reduced by higher baseline haemagglutinin inhibition titre (OR 0·11, 95% CI 0·04–0·27; p<0·0001) and increased by induction of an H3 haemagglutinin-specific CD4+ IL2-positive response (2·42, 1·05–5·62; p=0·037). Similar findings were seen in B/Vic and NY15 pH1N1 datasets, albeit with smaller sample sizes (appendix pp 18–20).

## Discussion

The findings of our study showed limited shedding, in vitro Cal09 replication, and low immunogenicity after administration of the 2016–17 LAIV in Gambian children, providing an explanation for the scant efficacy of this vaccine that was reported in a randomised controlled trial from neighbouring Senegal.^4^ After the switch to NY15, a significant increase in replication was seen, along with improved serum humoral and cellular immunogenicity. No competitive inhibitory effect of enhanced pH1N1 replication was recorded with H3N2 or B/Vic replication or immunogenicity. Our data also showed that shedding for a longer duration is important for immunogenicity and that viral replicative fitness should be considered alongside antigenicity when selecting vaccine strains. Our findings represent the first reported LAIV immunogenicity data from African children and make a case for further studies of LAIV efficacy in Africa. They are also of relevance to the use of LAIV in other settings.

In a study of Ann Arbor-backbone LAIV, improved shedding and haemagglutinin inhibition seroconversion was reported with an updated A/Slovenia/2015 pH1N1 strain.^17^ Parallel findings in two distinct cohorts of children—using two different LAIVs—provide strong support for Cal09 replicative fitness being culpable for the suboptimum pH1N1 LAIV effectiveness seen in recent years.^2^ Our finding that limited Cal09 shedding is unlikely to be attributable to pre-existing immunity further supports this result and argues against the notion that reduced LAIV effectiveness in the USA might have been due to repeated vaccination in previous years.^18^

In an earlier study using the Ann Arbor-backbone LAIV, pre-pandemic seasonal H1N1 shedding was found to be higher than for H3N2 or influenza B.^19^ Why pH1N1 Cal09 replication is impaired is uncertain. Haemagglutinin or neuraminidase residues must be the reason because the remaining six viral gene segments in LAIV are consistent between Cal09, NY15, and H3N2 strains. Differences in Cal09 haemagglutinin thermostability, sialic acid receptor binding, or pH sensitivity are potential explanations for the lower replication noted.^3^ These properties are important for replication in the human upper respiratory tract. In particular, the pH of the upper respiratory tract in children might be lower than that of adults^20^ and have a deleterious effect on replication of viruses with labile haemagglutinin. The pH1N1 virus first crossed into human beings in 2009 and has subsequently circulated as a human seasonal virus. During this time, changes in haemagglutinin stability and receptor binding properties might have adapted the virus to replicate better in the human upper respiratory tract.^21^ Thus, the more recent haemagglutinin from A/Michigan/45/2015-like viruses of 2015 could have conferred enhanced shedding to LAIV pH1N1 components.

We were able to mirror our findings in a primary human nasal epithelial cell model. These cells have a mildy acidic apical surface environment akin to that of the human upper respiratory tract. This strategy could be a practical method for assessing vaccine virus replication before strain choice. Because cell lines traditionally used to culture influenza viruses (eg, MDCK) might not truly reflect replication in the upper respiratory tract, these subtleties were previously underappreciated.^22^ Ultimately, a greater understanding of the viral genetic determinants of LAIV replicative fitness will be needed to select the best vaccine formulations.

Our study also emphasises the multifaceted nature of LAIV-induced immunity. Although seroresponse (the traditional correlate of protection after inactivated influenza vaccine) is modest, LAIV also induces mucosal IgA and T-cell responses. In our cohort, T-cell responses were elicited in a larger proportion of children than were mucosal or serum antibodies, showing the importance of assessing cellular immunity in LAIV studies. Using the 2017–18 LAIV formulation, a CD4+ IFNγ-positive or CD4+ IL2-positive T-cell response was seen in 55–68% of children to the influenza antigens tested, with approximately 80% of children showing a response to haemagglutinin or matrix and nucleoprotein (appendix p 18). LAIV provides protection in the absence of humoral immunity^23^ and T-cell-mediated immunity is thought to have an important role.^24^

Unlike serum antibody and T-cell responses, we did not see an increase in mucosal IgA responses with NY15. This finding is in keeping with results reported after one dose of the updated Ann Arbor-backbone LAIV,^17^ although a better response was seen after two doses. In a recent immunogenicity study of Nasovac-S in Bangladesh,^25^ unlike serum antibody, nasal pH1N1-specific IgA was induced despite scant Cal09 shedding. Furthermore, by contrast to seroconversion and T-cell responses, we noted no association between shedding and IgA responses. Taken together, these data suggest the mechanisms and requirements for serum antibody and mucosal IgA induction by LAIV could be distinct.

Our study has several limitations. Although the association between shedding and immunogenicity was a predefined exploratory objective in the larger randomised controlled trial our study was a part of, comparison of formulations containing Cal09 and NY15 was a post-hoc analysis made possible only because of the WHO-recommended update to pH1N1 in the 2017–18 formulation. Because we show an improvement in several shedding and immunogenicity endpoints with NY15, we are confident that our main conclusions are justified. Nevertheless, since our sample size was based on endpoints not reported here, the negative findings reported in some subanalyses should be interpreted with caution. Also, participants were vaccinated with one LAIV dose, in keeping with the prequalification license from WHO and the randomised controlled trials in Senegal^4^ and Bangladesh.^25^ Our findings, therefore, might not be generalisable to children in high-income countries who receive booster doses and yearly influenza vaccination. We were also unable to confirm viral shedding with an LAIV-specific RT-PCR in all participants because of lower sensitivity compared with the haemagglutinin-specific RT-PCR, which is important to fully exclude interference from wild-type circulating strains. However, by doing the study outside of the peak influenza season,^7^ undertaking clinical review at enrolment, and doing baseline RT-PCR screening for influenza virus, it is unlikely that our results were affected by wild-type influenza infections. Our shedding data at day 2 are also similar to those reported from Senegal using Nasovac-S (Cal09 19%, H3N2 48%, and influenza B 66%).^4^ Because we measured shedding with RT-PCR and not culture, we are unable to confirm to what degree shedding reflected viable viruses. Finally, an important unanswered question from our study is whether NY15 and related pH1N1 strains will result in improved LAIV effectiveness. Data from the 2017–18 UK season estimates the vaccine effectiveness (Ann Arbor-backbone LAIV) to be 90·3% against pH1N1 in children aged 2–17 years.^26^ However, owing to low-level circulation of pH1N1, the precision around this estimate was low (95% CI 16·4–98·9). Our findings suggest improved effectiveness can indeed be expected with the updated LAIV and, if so, would support wider use of LAIV in the prevention of influenza.
