# Supplementary material for: Effect of a Russian-backbone live-attenuated influenza vaccine with an updated pandemic H1N1 strain on shedding and immunogenicity among children in The Gambia: an open-label, observational, phase 4 study
Source: Lancet Respir Med. 2019 Aug;7(8):665–76. doi: 10.1016/S2213-2600(19)30086-4 (PMC6650545; doi:10.1016/S2213-2600(19)30086-4)
Supplement: Supplementary appendix [file mmc1.pdf]

THE LANCET
Respiratory Medicine

Supplementary appendix

This appendix formed part of the original submission and has been peer reviewed. We post it as supplied by the authors.

Supplement to: Lindsey BB, Jagne YJ, Armitage EP, et al. Effect of a Russian-backbone live-attenuated influenza vaccine with an updated pandemic H1N1 strain on shedding and immunogenicity among children in The Gambia: an open-label, observational, phase 4 study. *Lancet Respir Med* 2019; published online June 21. http://dx.doi.org/10.1016/S2213-2600(19)30086-4.

Table of Contents

Inclusion and Exclusion Criteria……………………………………………………………......Page 2
Summary of parent study profile…………………………………………………………………..Page 3
- Figure S1
- Table S1

Details of T-cell assays using flow cytometry……………………………………………..………...Page 5
- Table S2
- Figure S2
- Figure S3
- Table S3

Details of RT-PCR assays used in shedding endpoints……………………………………………...Page 8
- Table S4
- Figure S4
- Table S5

Correlation of nasopharyngeal viral loads………………………………………………………..Page 11
- Figure S5

Associations between baseline immune responses and shedding of LAIV strains…………………Page 12
- Table S6
- Table S7
- Table S8
- Figure S6

Further details of T-cell responses to LAIV………………………………………………………...Page 14
- Figure S7
- Figure S8
- Figure S9
- Figure S10
- Figure S11

Logistic regression analyses assessing the impact of shedding on immunogenicity to LAIV……...Page 16
- Table S9
- Table S10
- Table S11
- Table S12
- Table S13
- Table S14
- Table S15
- Table S16

Percentage of participants with T-cell responses to LAIV…………………………………………Page 20
- Figure S12

References……………………………………………………………………………….………..Page 21

<u>Inclusion and Exclusion Criteria</u>
Inclusion Criteria

- Healthy male or female child at least 24 months of age and less than 60 months of age at the time of study entry.
- Resident in the study area and with no plans to travel outside the study area during the period of subject participation.
- Informed consent for the study participation obtained from a parent (or guardian only if neither parent is alive or if guardianship has been legally transferred).
- Willingness and capacity to comply with the study protocol as judged by a member of the clinical trial team.

Exclusion Criteria

- Serious, active, medical condition, including but not limited to:
  - chronic disease of any body system
  - severe protein-energy malnutrition (weight-for-height Z-score of less than -3)
  - known genetic disorders, such as Down's syndrome or other cytogenetic disorder
- Active wheezing
- History of documented hypersensitivity to eggs or other components of the vaccine (including gelatin, sorbitol, lactalbumin and chicken protein), or with life-threatening reactions to previous influenza vaccinations.
- History of documented hypersensitivity to macrolide antibiotics
- History of Guillain-Barré syndrome.
- Receipt of aspirin therapy or aspirin-containing therapy within the two weeks before planned study vaccination.
- Any suspected or confirmed congenital or acquired state of immune deficiency including but not limited to primary immunodeficiencies including thymus disorders, HIV/AIDS, haematological or lymphoid malignancies.
- Any current immunosuppressive/immunomodulatory treatment or receipt of any such treatment within the six months preceding trial enrolment (for corticosteroids this is defined as a dose of prednisolone (or equivalent) of greater than 2mg/kg/day for one week or 1mg/kg/day for one month. The use of topical corticosteroids is not an exclusion criterion.
- The use of inhaled corticosteroids within the last one month.
- Receipt of an influenza vaccine within the past 12 months.
- Has any condition determined by investigator as likely to interfere with evaluation of the vaccine or be a significant potential health risk to the child or make it unlikely that the child would complete the study.
- Any significant signs or symptoms of an acute illness or infection including:
  - an axillary temperature of 38.0°C or above or documented fever of 38°C or above in the preceding 14 days.
  - Any acute respiratory infection within 14 days of enrolment visit.

Summary of parent study profile (ClinicalTrials.gov identifier NCT02972957)

The parent study is a phase 4, randomised controlled clinical vaccine trial: A Study of Intranasal Live Attenuated Influenza Vaccine Immunogenicity and Associations with the Nasopharyngeal Microbiome Among Children in The Gambia – The NASIMMUNE Study.

The sample size of the study and randomisation to LAIV vs unvaccinated arms was designed to document nasopharyngeal microbiome changes following LAIV compared to the control arm. Only participants receiving LAIV had samples taken for shedding and immunogenicity endpoints, therefore these represent data from a phase 4 observational study.

Children aged 24 – 59 months were recruited and randomised 1:1:1 to LAIV:LAIV:unvaccinated arms in both cohorts (target recruitment n = 110 in each group, total n = 330). This provided 90% power at alpha 0.05 to detect a 2-fold increase in density of relevant nasopharyngeal microbial taxa. The two LAIV groups followed identical study schedules other than the timing of an additional blood sample for exploratory immunological endpoints (at day 2 for one group and day 7 for the other), to minimise how many times each child was bled. In addition, in 2018, 35 children were recruited, given 1 dose of oral azithromycin, followed by the 2017-18 LAIV formulation 28 days later, for a pilot study to see if antibiotic therapy affected the interaction between LAIV and the nasopharyngeal microbiome. This group also had shedding and immunogenicity endpoints measured in an identical way to all other children given LAIV in the randomised groups. The summary study profile of the parent study is below (figure S1).

Data presented from the 2017-18 LAIV cohort therefore include data from n = 32 (of the total n = 126) who received azithromycin 28 days prior to LAIV. Shedding and immunogenicity endpoints from this group were not significantly different from those who did not received azithromycin (Table S1), therefore data are presented and analysed together.

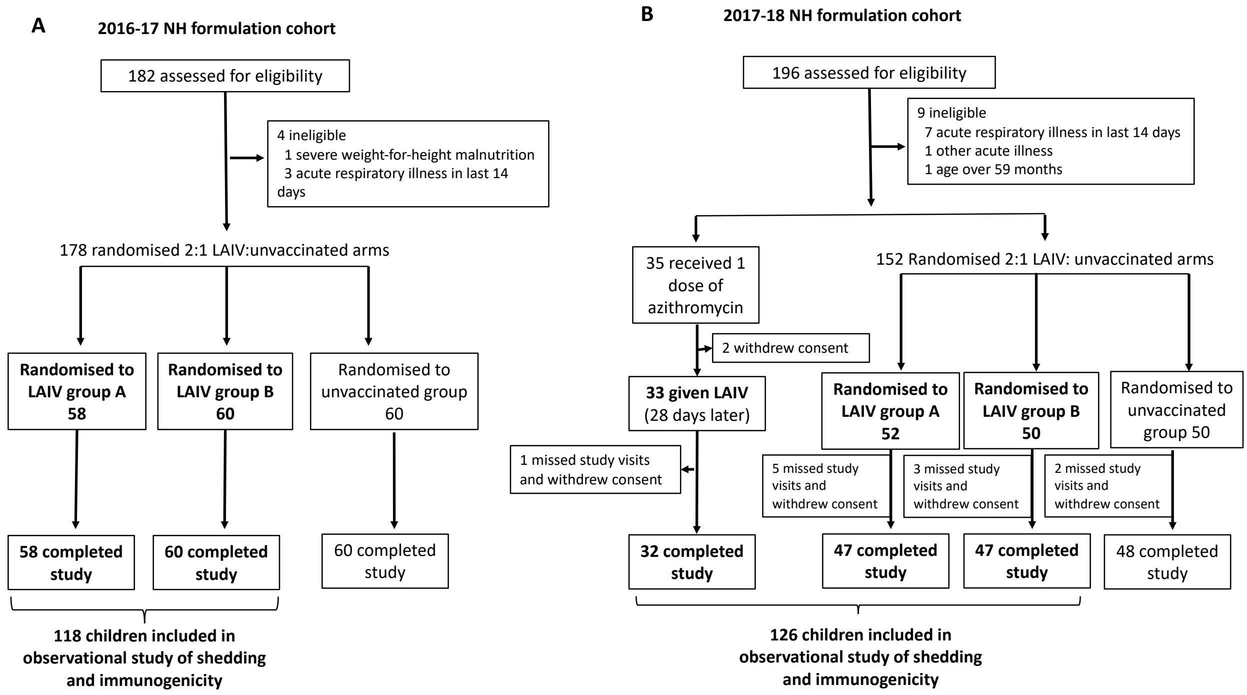

Figure S1. Trial profile of parent study. Participants given LAIV are in bold and correspond to numbers presented in Figure 1.

Shedding and immune responses	2017-18 cohort given azithromycin 28 days prior to LAIV (n = 32) % and 95% confidence interval	2017-18 cohort: other (n = 94) % and 95% confidence interval	p value from Fisher's exact test (unadjusted)
Children shedding pH1N1 at day 2	65.62 (46.81-81.43)	62.77 (52.18-72.52)	0.8847
Children shedding H3N2 at day 2	65.62(46.81-81.43)	64.89 (54.36-74.46)	1
Children shedding B/Vic at day 2	78.12 (60.03-90.72)	70.21 (59.9-79.21)	0.4762
Children shedding pH1N1 at day 7	46.88 (29.09-65.26)	53.19 (42.61-63.56)	0.7916
Children shedding H3N2 at day 7	34.38 (18.57-53.19)	30.85 (21.73-41.22)	0.8529
Children shedding B/Vic at day 7	50.0 (31.89-68.11)	46.81 (36.44-57.39)	0.8758
Seroconversion pH1N1	15.62 (5.28-32.79)	20.21 (12.63-29.75)	0.7751
Seroconversion H3N2	25.0 (11.46-43.4)	28.72 (19.86-38.98)	0.884
Seroconversion B/Vic	28.12 (13.75-46.75)	22.34 (14.39-32.1)	0.6507
pH1 HA IgA response	19.35 (7.45-37.47)	19.54 (11.81-29.43)	1
H3 HA IgA response	10.34 (2.19-27.35)	18.89 (11.41-28.51)	0.3602
B/Vic HA IgA response	25.81 (11.86-44.61)	30.77 (21.51-41.32)	0.7678
CD4+IFN-gamma+ pH1 HA response	46.43 (27.51-66.13)	46.25 (35.03-57.76)	1
CD4+IFN-gamma+ H3 HA response	14.29 (4.03-32.67)	36.25 (25.79-47.76)	0.05321
CD4+IFN-gamma+ B/Vic HA response	46.43 (27.51-66.13)	31.25 (21.35-42.59)	0.2234

Table S1. Comparison of shedding and immunogenicity endpoints in individuals in the 2017-18 LAIV cohort given one dose of azithromycin 28 days prior to LAIV with those who did not receive any antibiotics.

Details of T-cell assays using flow cytometry

Marker	Fluorochrome	Manufacturer	Clone
CD4	PerCP-Cy5.5	Biolegend	RPA-T4
CD8	FITC	Biolegend	RPA-T8
IL-2	PE	Biolegend	MQ1-17H12
IFN-γ	APC	Biolegend	B27
Viability dye	Live/dead® Aqua	Biolegend	NA

Table S2. Antibodies used in flow cytometry assays to detect influenza-specific T-cell response. IFN-γ = interferon-gamma, IL-2 = interleukin-2.

Fresh whole blood was stimulated with pooled overlapping 15-18-mer peptides (Sigma Aldrich) corresponding to influenza A pH1 HA, H3 HA, Matrix and Nucleoprotein, as well as influenza B (Victoria) lineage HA, Matrix and Nucleoprotein in 2018. Peptides were designed based on exact sequences of vaccine strains (i.e. seasonal influenza HAs and master donor virus Matrix and Nucleoprotein). This included Cal09 and NY15-specific peptide sets used in children given Cal09 or NY15 respectively. Stimulations of 200μl of fresh whole blood were set up on the day of sample collection. In addition to relevant influenza peptides (along with co-stimulatory antibodies anti-CD28 and anti-CD49), all samples were also stimulated with Staphylococcal Enterotoxin B (SEB, positive control) and anti-CD28 and anti-CD49 alone (negative control). As peptides are originally reconstituted in dimethyl sulfoxide (DMSO), the same percentage of DMSO was added to negative controls as contained in the peptide stimulated conditions, to ensure that any inhibitory effect was consistent across conditions. Stimulations were incubated at 37°C 5% CO_2 for 2 hours, after which protein-transport inhibitor Brefeldin A (BD Golgiplug) was added to all tubes and incubations continued for a further 16 hours. After overnight incubation, blood was stained with live-dead Aqua viability dye to discriminate live and dead cells, followed by surface staining with anti-CD4 and anti-CD8 antibodies. Cells were lysed, permeabilised and stained intra-cellularly with anti-IFN-γ and anti-IL-2 antibodies.

Cells were acquired on an LSR Fortessa flow cytometer, based on pre-defined voltage settings kept consistent across all time points. Daily quality control and calibration checks were undertaken on the flow cytometer to ensure results from different days were as comparable as possible. At least 200,000 live, lymphocyte events were recorded for each sample.

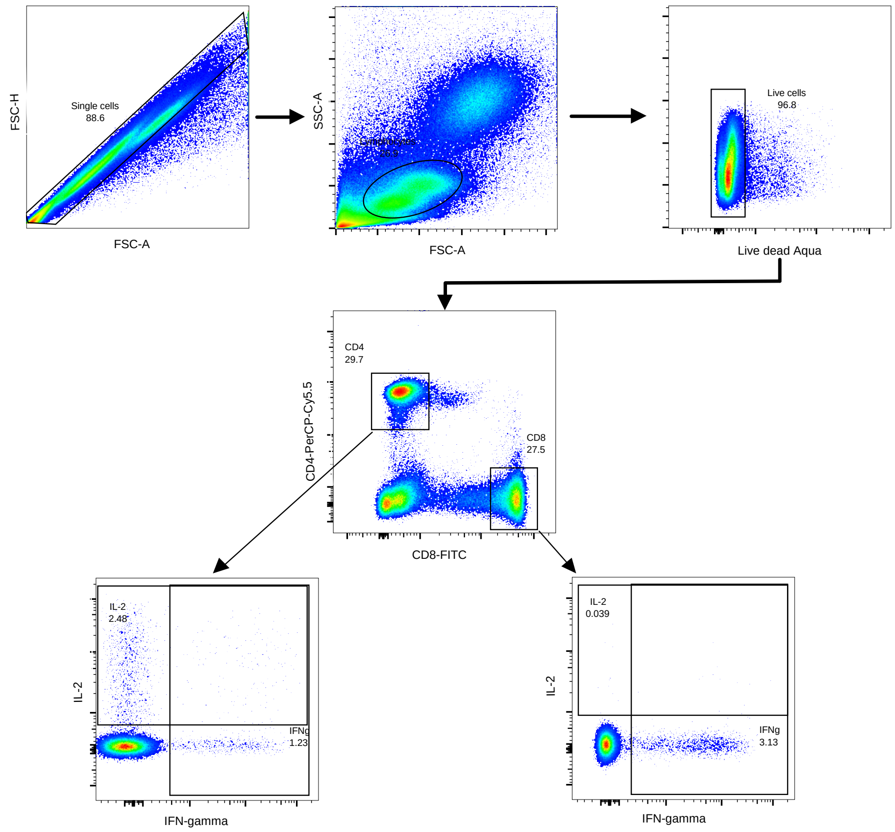

Figure S2. Gating strategy for detection of IFN-γ and IL-2 in CD4+ and CD8+ T-cells. Displayed is from a sample following overnight stimulation with Staphylococcal Enterotoxin B (SEB).

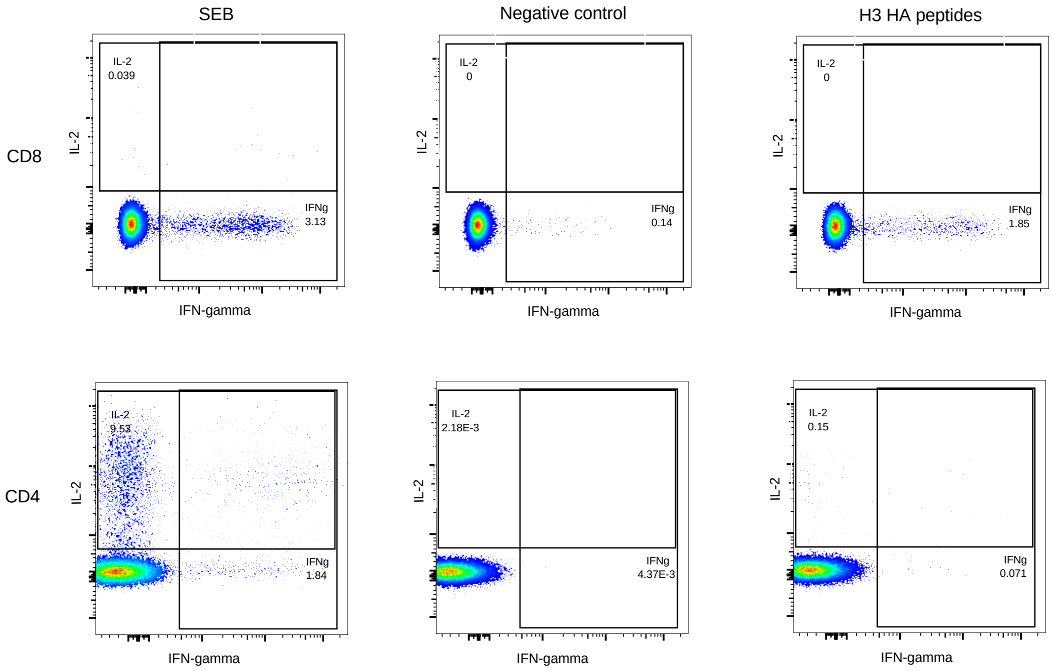

Figure S3. CD8+ and CD4+ T-cell responses (IFN-γ and IL-2) following stimulation with Staphylococcal Enterotoxin B (SEB), negative control (co-stimulatory antibodies anti-CD28 and anti-CD48 alone) and an overlapping peptide pool matched to H3 haemagglutinin protein (A/H3N2 Hong Kong/4801/2014).

	2017 data	2018 data
**CD4 IFN-γ**	0.009	0.014
**CD4 IL-2+**	0.015	0.016
**CD8 IFN-γ+**	0.047	0.110
**CD8 IL-2+**	0.005	0.014
**CD4 IFN-γ+IL-2+**	0.003	0.003
**CD4 IFN-γ+IL-2-**	0.007	0.013
**CD4 IFN-γ-IL-2+**	0.013	0.015

Table S3. Thresholds calculated based on the distribution of negative values after background subtraction, below which a positive value was also considered a null response.[1-3] IFN-γ = interferon-gamma, IL-2 = interleukin-2.

Details of RT-PCR assays used in shedding endpoints

(i) RT-PCR conditions

Equine Arteritis Virus was added as an internal control followed by RNA extraction from NPS (260µl) using the QIAmp Viral RNA Mini Kit (Qiagen). Extracted RNA was reverse transcribed using 4X TaqMan™ Fast Virus 1-Step Master Mix (ThermoFisher) at 50˚C for 15 minutes, before denaturation at 95˚C for 60 seconds, followed by 50 cycles of amplification at 95˚C for 10 seconds and 60˚C for 30 seconds using a LightCycler® 96 (Roche). Cycle threshold (ct) values and positive sample calling were determined using automated LightCycler® software. Positive (seasonal influenza viruses) and negative (RNAse-free water) controls were included on each plate. All positive controls were consistently within two ct values.

Name	Target	Label	Sequence
**HA-specific RT-PCR#**			
H1-Sw-1306F	pH1N1 HA	-	TGG ACT TAC AAT GCC GAA CT
H1-Sw-1423R	pH1N1 HA	-	CAG CCG TTT CCA ATT TCC TT
H1-sw-1357P2	pH1N1 HA	TXR-BHQ2	GAC TAC CAC GAT TCA AAT GTG AAG AAC T
H3-1541F1	H3N2 HA	-	GAT GTR TAC AGA GAT GAA GCA TTA AAC A
H3-1600R	H3N2 HA	-	TAG GAT CCA ATC TTT GTA CCC TGA CTT
H3-1571P1	H3N2 HA	YY-BHQ1	AGC TCA ACT CCC TTG ATC TGG AAY CGG
INFB-HA-444F	Influenza B HA	-	ACC CTA CAR AMT TGG AAC YTC AGG
INFB-HA-524R	Influenza B HA	-	ACR GCC CAA GCC ATT GTT G
INFB-Vic499Pr	Influenza B Victoria lineage HA	Atto532-MGBFQ	ATC CGT TTC CAT TGG TAA
EAV-2043F	Equine Arteritis Virus (IC)	-	CTG TCG CTT GTG CTC AAT TTA C
EAV-2193R	Equine Arteritis Virus (IC)	-	AGC GTC CGA AGC ATC TC
EAV 2102P-2	Equine Arteritis Virus (IC)	TXR-BHQ2	TGC AGC TTA TGT TCC TTG CAC TGT GTT C
**Seasonal influenza internal gene RT-PCR#**			
INFAM-sense	Influenza A matrix	-	AAG ACC AAT CCT GTC ACC TCT GA
INFAM-sense3	Influenza A matrix	-	AAG ACC AAT CTT GTC ACC TCT GA
INFAM-sense4	Influenza A matrix	-	AAG ACC AAT TCT GTC ACC TCT GA
INFAM A-sense	Influenza A matrix	-	CAA AGC GTC TAC GCT GCA GTC C
INFAM- probe2-EDQ	Influenza A matrix	FAM-EDQ	TTT GTG TTC ACG CTC ACC GTG CC
INFB-NS779F	Influenza B NS gene	-	GTC TTA ATG AAG GAC ATT CAA AGC C
INFB-NS886R	Influenza B NS gene	-	TAA AGT TCT TCC GTG ACC AGT CTA
INFB 848P	Influenza B NS gene	YY-BHQ1	GTC AAG AGC ACC GAT TAT CAC CAG AAG AG
**LAIV-specific internal gene RT-PCR***			
MLenF427	Influenza A	-	GCC TTT GGC CTG GTA TGT G
MLenR488	Influenza A	-	CTA TGA GAC CTA TGC TGG GAG TCA
MLenP447	Influenza A	FAM-BHQ1	AAC CTG TGA ACA GAT TG
NSRussiaF377	Influenza B NS gene	-	GAA AGT GCC TTG ATG ACA TA
NSRussiaR461	Influenza B NS gene	-	TCT TTG TTG TTC ATG TCC CTC AAT
NSRussiaP426r	Influenza B NS gene	Atto532-BHQ1	TGG GTC ATC AAC ATT TTC CGG TTC

Table S4. Primer and probe sequences used in reverse-transcriptase polymerase chain reaction (RT-PCR) assays. #Routinely used in surveillance of influenza at the National Institute for Public Health and the Environment, Bilthoven, the Netherlands, which is one of the two locations of the National Influenza Centre in which these tests are performed under ISO 15189 accreditation for medical laboratories.[4] *Influenza A assays for LAIV-specific RT-PCR were based on previously published primers and probes that distinguish seasonal influenza A from LAIV influenza A master donor virus.[5] Influenza B assays for LAIV-specific RT-PCR were modified from previously published primers and probes for seasonal influenza B and incorporated key changes to match the B/Russia/69 sequence (which represents a close match the

Nasovac-S influenza B master donor virus) and differentiate from current circulating strains. IC – internal control. HA = haemagglutinin.

(ii) <u>Details of quantitative RT-PCR assay used with the 2017-18 cohort:</u>

A standard curve was included on each plate with two replicates of LAIV dilutions from $1{:}10^3$ to $1{:}10^6$, with known log10 50% Egg Infectious Doses/ml (EID/ml). Testing during assay validation showed continued linearity up to a 1:10 dilution. Median RT-PCR efficiency (based on standard curve) was 98% (range 88-110%). Median standard curve R^2 was 0.998 (range 0.977-1). 95% LOD was determined using a serial dilution (10 replicates) of 2017-18 formulation vaccine (figure S3). LOD for pH1N1, H3N2 and Influenza B PCRs were 1.11 (95% CI 0.89-1.22), 0.91 (0.61-1.09) and 0.73 (0.5-0.85) EID/ml respectively (Figure S4 below).

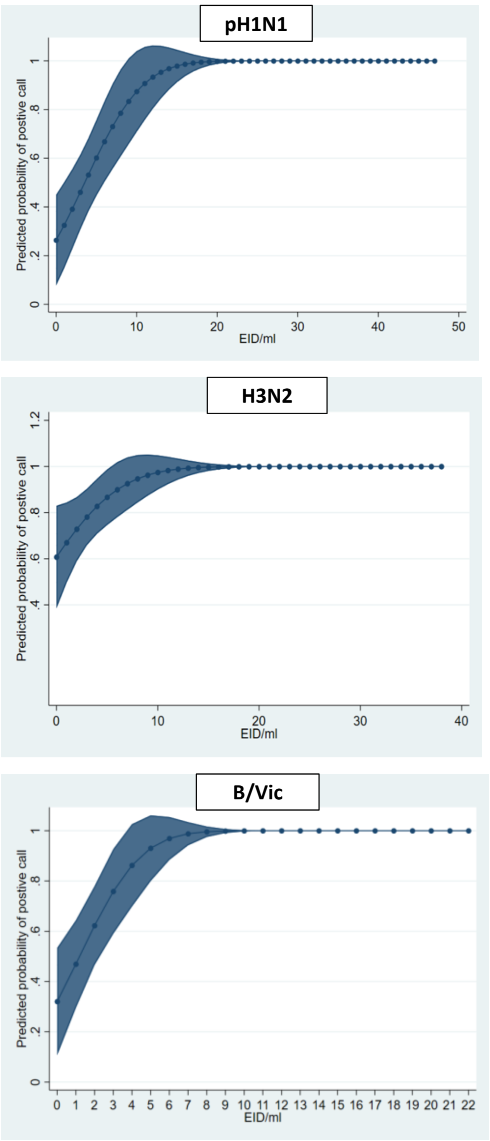

Figure S4. 95% limit of detection (LOD) for pH1N1, H3N2 and B/Vic RT-PCR using HA-specific primer and probes sets. Calculated using a 2-fold serial dilution of 10 LAIV replicates from $1{:}4 \times 10^6$ to $1{:}6.4 \times 10^7$ (after an initial 10-fold three-replicate dilution series from $1{:}10^3$ to $1{:}10^9$ to estimate the LOD for each RT-PCR). Displayed are predicted probability (and 95% confidence interval) of a positive reaction at each log10 50% egg infectious dose/ml (EID/ml) calculated using Stata 12.1 (Probit model).[6] LOD for pH1N1, H3N2 and Influenza B PCRs were 1.11 (95% CI 0.89-1.22), 0.91 (0.61-1.09) and 0.73 (0.5-0.85) EID/ml respectively.

(iii) LAIV-specific RT-PCR:

A 10-fold dilution series ($1{:}10^2 – 1{:}10^8$) of seasonal influenza strains and LAIV were tested with seasonal influenza and LAIV-specific sets (table S4). LAIV-specific assays detected LAIV but not seasonal influenza strains (pH1N1, H3N2 and B/Victoria), whereas as expected, seasonal influenza assays detected both. Despite optimisation of assay conditions, the maximum LAIV dilution detected by LAIV sets was at least one log10 lower than that detected by HA-specific sets (Table S5).

Primer/probe set (target)	Highest LAIV dilution detected*
LAIV influenza A (matrix)	$1{:}10^6$
LAIV influenza B (NS)	$1{:}10^5$
Seasonal influenza A (matrix)	$1{:}10^7$
Seasonal influenza B (NS)	$1{:}10^6$
pH1N1 (HA)	$1{:}10^7$
H3N2 (HA)	$1{:}10^7$
B/Vic (HA)	$1{:}10^8$

Table S5. Ability of LAIV and seasonal influenza primer/probe sets to detect LAIV strains. *results from 10-fold dilution series from $1{:}10^2$ to $1{:}10^8$ run in quadruplicate. Displayed are highest dilutions at which at least one positive reaction was seen. Haemagglutinin (HA)-specific sets appeared to be more sensitive than LAIV (matrix and NS1)-specific sets. Of note the influenza A matrix sets should amplify influenza A LAIV master donor virus from both pH1N1 and H3N2 vaccine strains, thus approximately double the amount of virus should be available as a target compared to influenza B internal gene sets or any HA-specific sets.

Correlation of nasopharyngeal viral loads

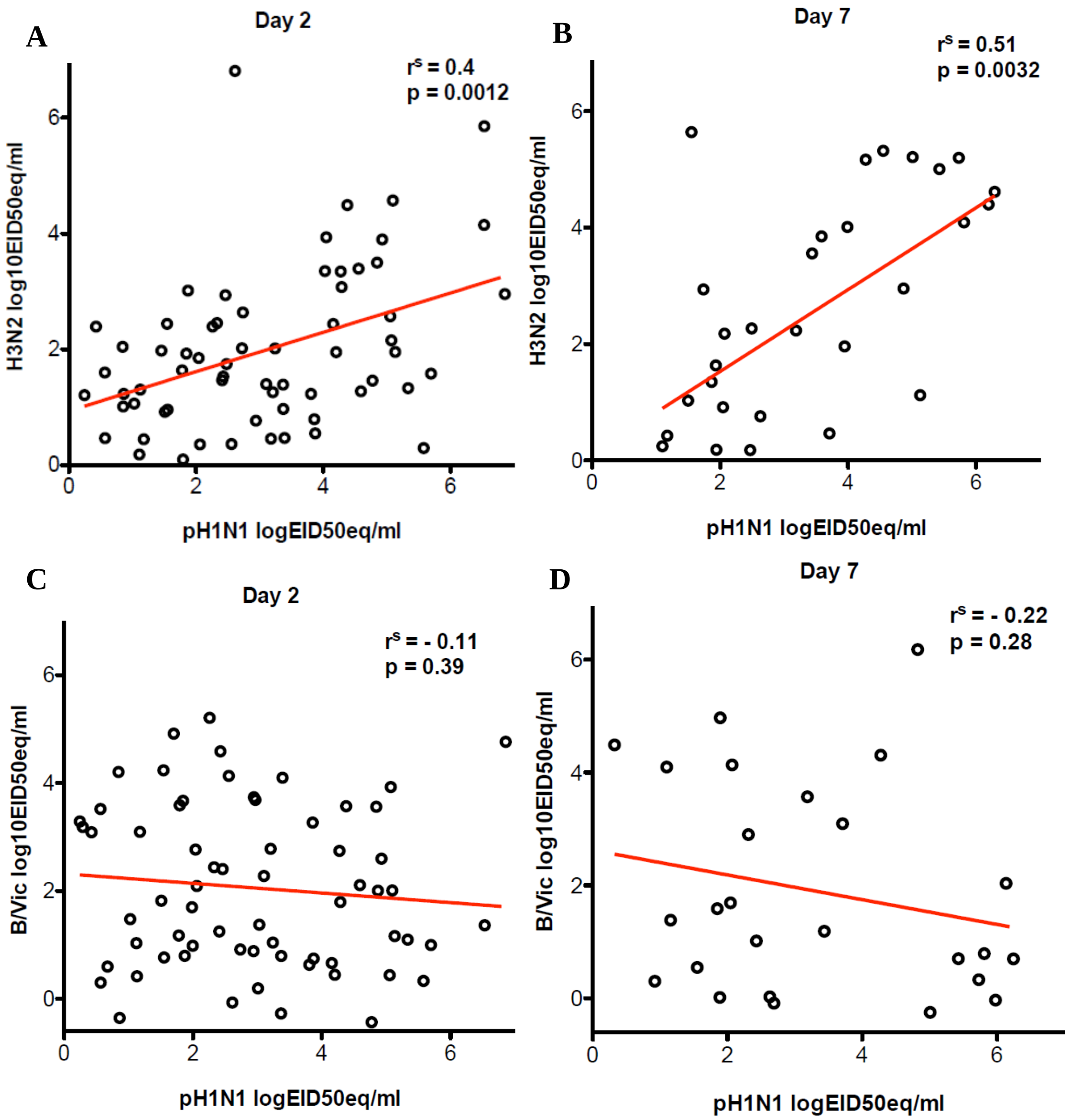

Figure S5. **Correlation of nasopharyngeal viral loads in children given 2017-18 formulation between day 2 pH1N1 and H3N2.** Correlation of nasopharyngeal viral loads in children given 2017-18 formulation between day 2 pH1N1 and H3N2 **(A)**, day 7 pH1N1 and H3N2 **(B)**, day 2 pH1N1 and B/Vic **(C)** and day 7 pH1N1 and B/Vic strains **(D)**. r^s = Spearman's rank order coefficient. All displayed p values are Bonferroni-adjusted for multiplicity within each group of analyses.

Associations between baseline immune responses and shedding of LAIV strains

		Univariable		
		**OR**	**95% CI**	**p value**
Age (in months)		0.99	0.93-1.05	0.779
Sex		1.08	0.51-2.30	0.884
Z-score (weight-for-height)		1.32	0.78-2.22	0.343
Baseline seropositivity (HAI titre)		1.25	0.48-3.22	0.684
Baseline IgA (% of total IgA)*	2nd tertile	3.33	0.47-23.72	0.276
	3rd tertile	2.06	0.29-14.82	0.515
Baseline CD4+ T-cell MNP IFN-γ response		0.27	0.04-1.74	0.214
Baseline CD8+ T-cell MNP IFN-γ response		0.31	0.05-2.11	0.277

Table S6. No association between baseline immunity and shedding at day 2 of 2016-17 LAIV pH1N1 (Cal09) strain in univariable logistic regression. OR = odds ratio. HAI = haemagglutination inhibition. MNP = matrix and nucleoprotein. IFN-γ = interferon gamma. *1st tertile used as reference for calculating ORs.

**Baseline immune response**		**Strain**	**Univariable**			**Adjusted for baseline HAI titre**		
			OR	95% CI	p value	OR	95% CI	p value
**Influenza-specific IgA (% of total)**	2nd tertile	*pH1N1 NY15*	*0.6*	0.27-1.32	0.250	-		-
	3rd tertile		0.47	0.19-1.13	0.131	-		-
**Influenza-specific IgA (% of total)†**	2nd tertile	H3N2	0.63	0.35-1.14	0.169	0.99	0.76-1.30	0.988††
	3rd tertile		**0.31**	**0.17-0.56**	**<0.001**	0.68	0.35-1.34	0.311††
**Influenza-specific IgA (% of total)†**	2nd tertile	B/Vic	0.69	0.34-1.38	0.340	-		-
	3rd tertile		0.60	0.31-1.17	0.178	-		-
**CD4+ T-cell A/MNP IFN-γ response**		pH1N1 NY15	0.39	0.11-1.34	0.07	-		-
**CD8+ T-cell A/MNP IFN-γ response**		pH1N1 NY15	0.23	0.03-2.12	0.24	-		-
**CD4+ T-cell A/MNP IFN-γ response†**		H3N2	**0.40**	**0.23-0.71**	**0.007**	0.52	0.28-1.00	0.069††
**CD8+ T-cell A/MNP IFN-γ response†**		H3N2	0.75	0.39-1.44	0.43	-		-
**CD4+ T-cell B/MNP IFN-γ response***		B/Vic	0.33	0.11-1.00	0.082	-		-
**CD8+ T-cell B/MNP IFN-γ response***		B/Vic	No baseline responses seen					

Table S7. Impact of pre-existing IgA and T-cell immunity on probability of shedding following LAIV. †Combined 2017 & 2018 data. ††Year included in multivariable model. 1st tertile used as reference in IgA analyses. *Influenza B T-cell responses only available from 2018 cohort. No CD8+ T-cell responses to vaccine antigen-matched influenza B master donor virus matrix or nucleoprotein seen (above calculated thresholds defining a true response) in baseline samples. OR = odds ratio. MNP = matrix and nucleoprotein. HAI = haemagglutination inhibition. IFN-γ = interferon gamma.

	pH1N1		H3N2		B/Vic	
Baseline (reciprocal) HAI titre	2016-17 (Cal09)	2017-18 (NY15)	2016-17	2017-18	2016-17	2017-18
<10	10/79	58/64	21/28	46/56	78/93	57/72
7.1	-	-	-	-	-	1/1
10	-	-	-	1/1	-	-
20	-	0/1	-	-	-	-
28.3	-	-	1/2	-	-	-
40	0/1	1/4	3/4	7/8	2/2	1/2
56.6	-	-	1/3	-	1/1	0/1
80	1/14	4/9	7/22	5/12	6/7	2/4
113.1	1/3	-	2/3	0/1	-	-
160	3/15	7/15	14/34	7/20	5/7	12/16
226.3	0/2	3/4	1/3	3/3	1/2	5/9
320	1/4	4/17	2/13	10/16	2/5	7/11
452.6	-	0/3	0/1	1/2	0/1	1/2
640	-	3/8	1/4	2/7	-	4/7
905.1	-	-	1/1	-	-	1/1
1280	-	0/1	-	-	-	-

Table S8. Number of children shedding LAIV strains at day 2 post-LAIV. Denominator in each cell is the number of participants with the respective Haemagglutination inhibition (HAI) titre at baseline.

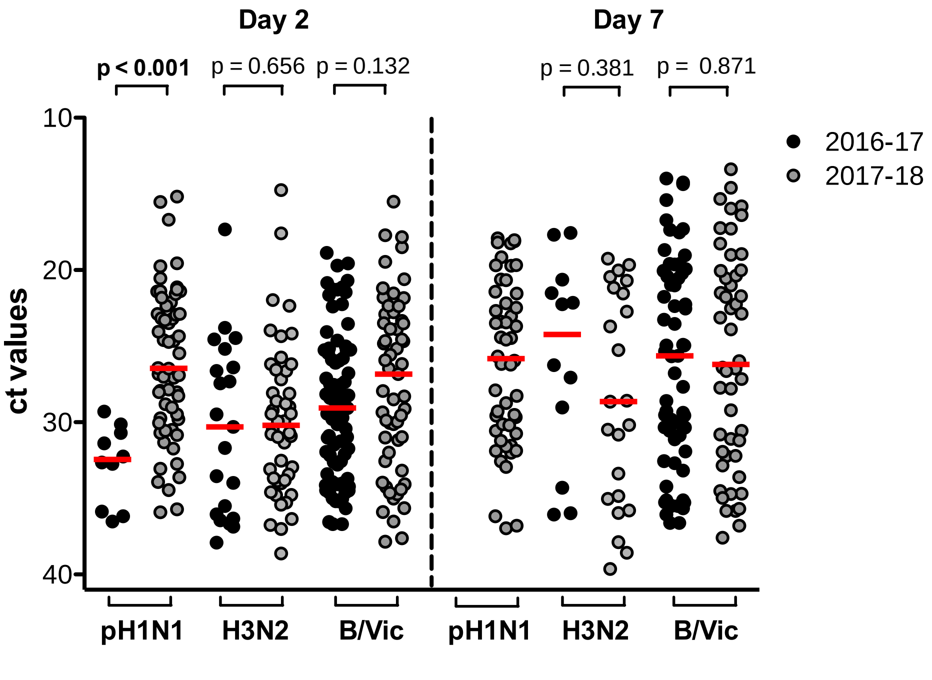

Figure S6. Cycle threshold (ct) values from reverse-transcriptase polymerase chain reaction for each strain, as a marker of viral load in the nasopharynx, in children seronegative at baseline. Red bars indicate median ct values. Note lower ct values indicate higher viral loads. All displayed p values are Bonferroni-adjusted for multiplicity within each group of analyses. No comparison of day 7 pH1N1 shedding possible as no pH1N1 was detected in samples at day 7 with the 2016-17 LAIV.

Further details of T-cell responses to LAIV

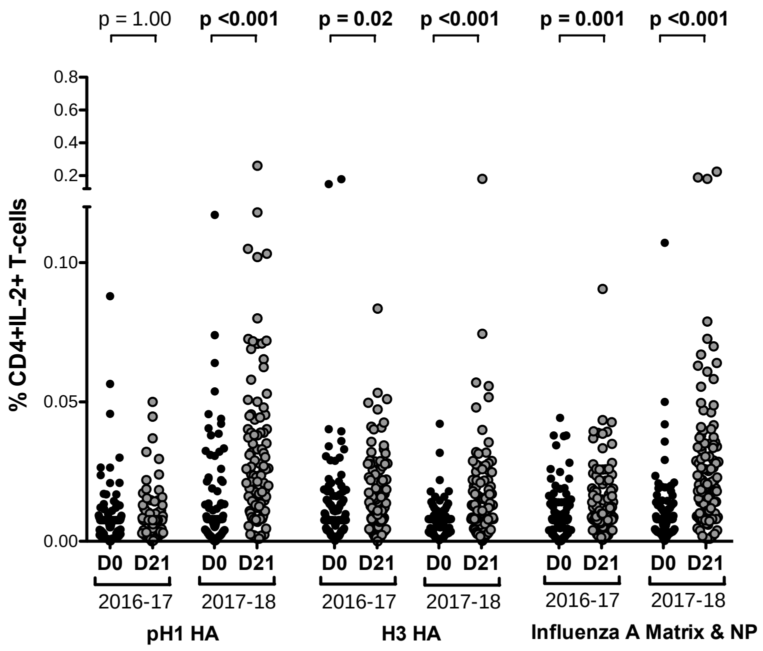

Figure S7. CD4+IL2+ T-cell responses to influenza A antigens using the 2016-17 and 2017-18 LAIV formulations. Significant increase from day 0 (D0) to day 21 (D21) of IL-2+ CD4+ T-cell responses to pH1 haemagglutinin (HA) seen only using 2017-18 formulation. CD4+IL-2+ T-cell responses to H3 HA, as well as matrix and nucleoprotein (NP) antigen seen using both formulations. IFN-γ = interferon gamma. IL-2 = interleukin 2. P values are Bonferroni-adjusted for multiple comparisons.

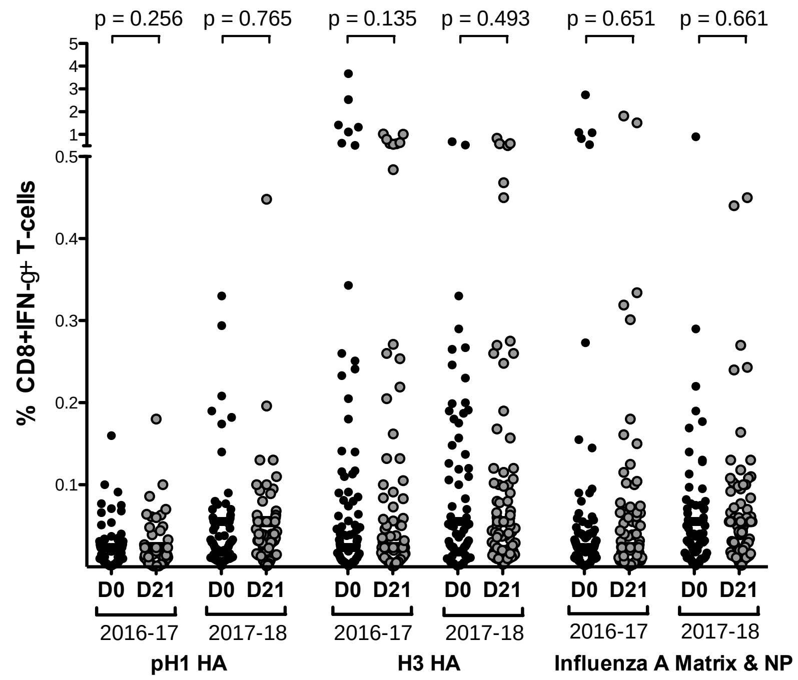

Figure S8. CD8+ IFN-γ+ T-cell responses to influenza A antigens using the 2016-17 and 2017-18 LAIV formulations. No significant increase from day 0 (D0) to day 21 (D21) seen to pH1 haemagglutinin (HA), H3 HA or matrix and nucleoprotein (NP) antigens seen using either formulation. IFN-γ = interferon gamma.

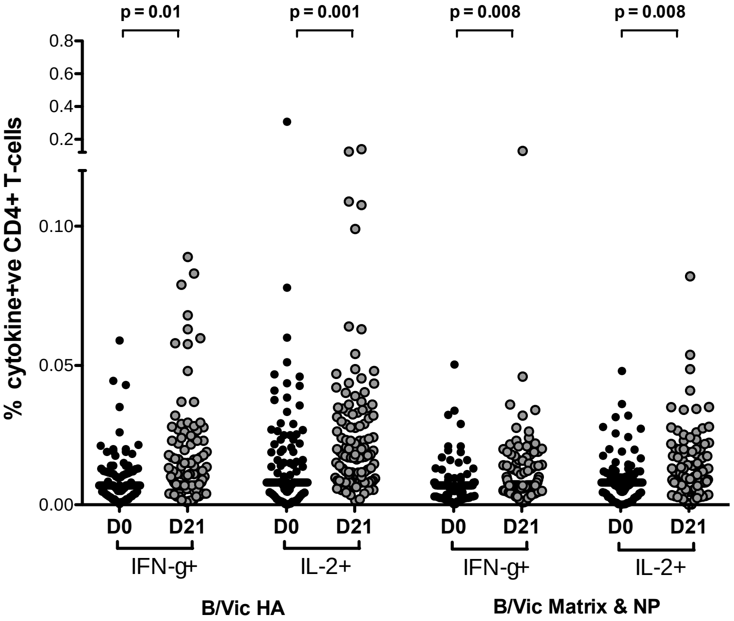

Figure S9. Induction of CD4+ T-cell responses to influenza B antigens using the 2017-18 LAIV formulation. Significant increase from day 0 (D0) to day 21 (D21) of IFN-γ+ and IL-2+ CD4+ T-cell responses to influenza B Victoria lineage (B/Vic) haemagglutinin (HA), as well as matrix and nucleoprotein (NP) antigen from Russian-backbone LAIV. IFN-γ = interferon gamma. IL-2 = interleukin 2. P values are Bonferroni-adjusted for multiple comparisons.

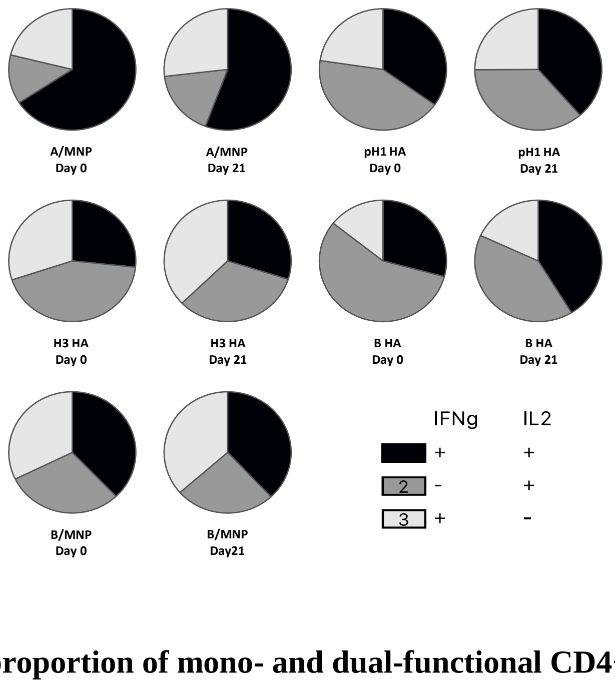

Figure S10. The proportion of mono- and dual-functional CD4+ T-cell responses to influenza antigens tested. No difference seen between baseline and day 21. Influenza B HA and matrix and nucleoprotein (MNP) data are from 2018 only, whereas others are combined 2017 and 2018 data. Significance between proportions of mono- and dual- functional responses across timepoints tested using the Permutation test (SPICE V6.0). IFNg = interferon gamma. IL-2 = interleukin 2.

Logistic regression analyses assessing the impact of shedding on immunogenicity to LAIV

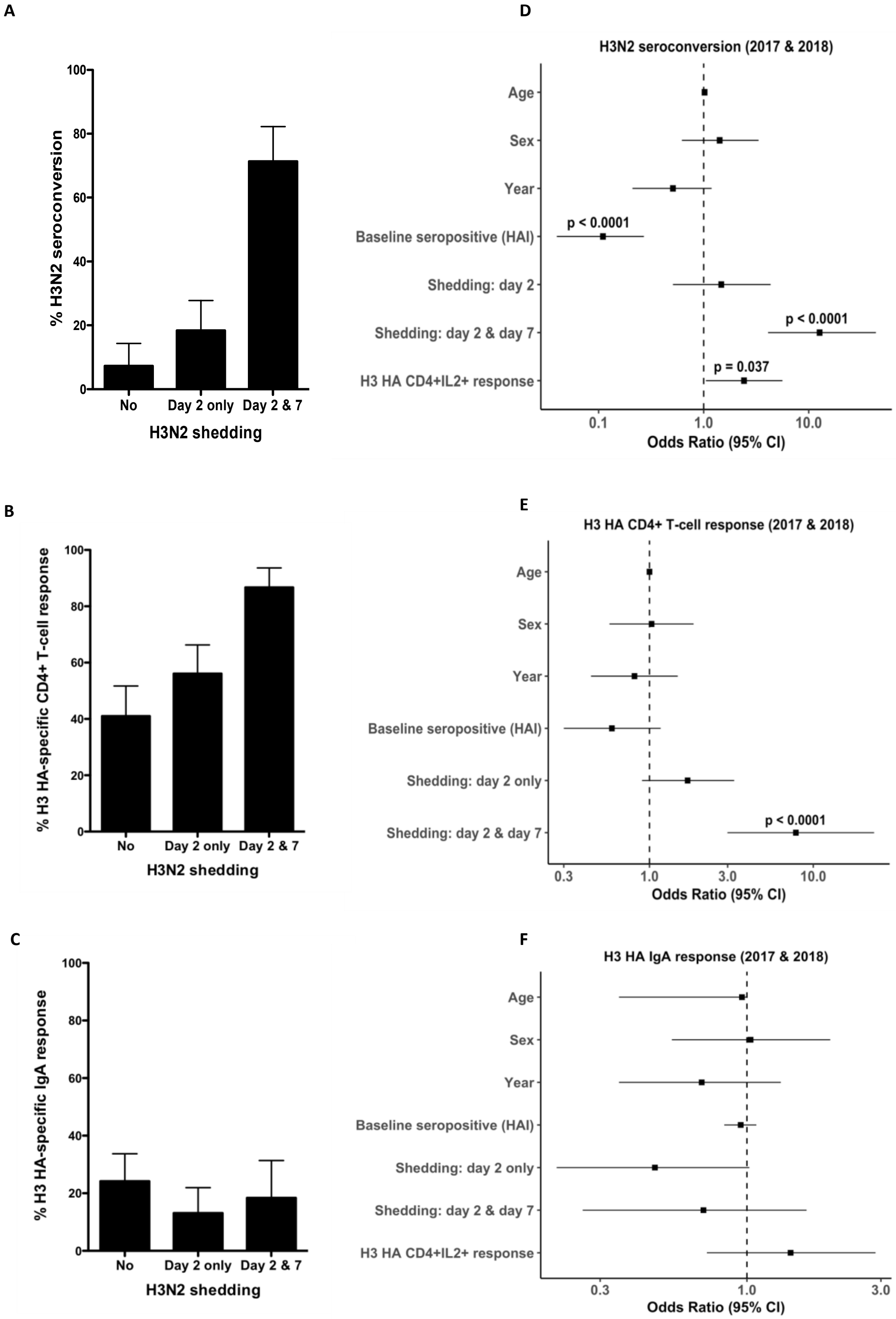

Figure S11. The impact of nasopharyngeal shedding on immune responses to LAIV. Percentage of children with **(A)** seroconversion, **(B)** 2-fold rise in haemagglutinin (HA)-specific CD4+ T-cell response (IFN-γ and/or IL-2) and **(C)** 2-fold rise in influenza HA-specific mucosal Immunoglobulin A (IgA) with each shedding category (no shedding, shedding at day 2 only,

shedding at day 2 and day 7), using H3N2 data as an example (combined 2016-17 and 2017-18 data). Children without complete T-cell data (24/244) were excluded. The small number of children with shedding detected only at day 7 but not day 2 (12/244) were also excluded from the analysis. Odds ratio and 95% confidence intervals (CI) from multivariable logistic regression assessing the impact of shedding and other variables on **(D)** seroconversion, **(E)** CD4+ T-cell induction**. (F)** Odds ratio and 95% CI from univariate logistic regression assessing the impact of shedding on IgA response to H3N2. Odds ratio for shedding categories are calculated using no shedding as the reference.

**H3N2 seroconversion (2017 & 2018)**		Univariable			Multivariable		
		OR	95% CI	p value	OR	95% CI	p value
Year		1.37	0.75 - 2.53	0.315	0.51	0.21 - 1.19	0.128
Age		0.99	0.95 - 1.02	0.491	1.02	0.98 - 1.08	0.239
Sex		1.15	0.63 - 2.13	0.648	1.42	0.62 - 3.33	0.406
Baseline seropositive (HAI titre)		**0.1**	**0.05 - 0.2**	**<0.0001**	**0.11**	**0.04 - 0.27**	**<0.0001**
Shedding	Day 2 only	2.43	0.96 - 6.7	0.068	1.45	0.51 - 4.34	0.494
	Day 2 and Day 7	**26.7**	**10.29 - 78.52**	**<0.0001**	**12.69**	**4.1 - 43.6**	**<0.0001**
H3 HA CD4+IFN-γ+ response		**1.71**	**0.92 - 3.19**	**0.086**	-	-	-
H3 HA CD4+IL-2+ response		**4.95**	**2.61 - 9.7**	**<0.0001**	**2.42**	**1.05 - 5.62**	**0.037**

Table S9. Impact of shedding on seroconversion to H3N2. Univariable and multivariable logistic regression using n = 210 children. Excludes 24/244 participants with missing T-cell data and 12/224 with shedding only detected at day 7 (2 children had both T-cell data missing and shedding only at day 7). Shedding coded as one variable with three levels (no shedding, day 2 only shedding, day 2 and day 7 shedding). H3 Haemagglutinin (HA) CD4+IL-2+ response taken forward to multivariable analysis due to overlap between this and the CD4+IFN-γ response. No interactions observed between variables included in the multivariable analysis. OR = odds ratio, CI = confidence interval, HAI = haemagglutination inhibition

**H3 HA CD4+IFN-γ and/or IL2 response (2017 & 2018)**		Univariable			Multivariable		
		OR	95% CI	p value	OR	95% CI	p value
Year		1.21	0.71 - 2.07	0.486	0.81	0.44 - 1.49	0.500
Age		0.99	0.96 - 1.02	0.533	1.00	0.97 - 1.03	0.947
Sex		1.05	0.61 - 1.79	0.866	1.03	0.57 - 1.86	0.915
Baseline seropositive (HAI)		**0.39**	**0.21 - 0.69**	**0.002**	0.59	0.3 - 1.17	0.134
Shedding	Day 2 only	1.84	1.00 - 3.44	0.053	1.71	0.9 - 3.29	0.102
	Day 2 and Day 7	9.38	3.8 - 26.85	**<0.0001**	**7.83**	**2.99 - 23.5**	**<0.0001**

Table S10. Impact of H3N2 shedding on H3 HA-specific CD4+ T-cell response (IFN-γ and/or IL-2). Univariable and multivariable logistic regression using n = 210 children. Excludes 24/244 participants with missing T-cell data and 12/224 with shedding only detected at day 7 (2 children had both T-cell data missing and shedding only at day 7). Shedding coded as one variable with three levels (no shedding, day 2 only shedding and day 2 and day 7 shedding). No interactions observed between variables included in the multivariable analysis. OR = odds ratio, CI = confidence interval, HAI = haemagglutination inhibition

H3 HA IgA response (2017 & 2018)		Univariable		
		OR	95% CI	p value
Year		0.69	0.35 - 1.32	0.268
Age		0.96	0.93 - 1.00	0.071
Sex		1.03	0.54 - 1.98	0.928
Baseline seropositive (HAI)		0.95	0.83 - 1.08	0.413
Shedding	Day 2	0.47	0.21 - 1.02	0.062
	Day 2 and Day 7	0.70	0.26 - 1.63	0.426
H3 HA CD4+IFNg+ response		1.02	0.50 - 2.06	0.946
H3 HA CD4+IL2+ response		1.43	0.72 - 2.87	0.304

Table S11. Impact of H3N2 shedding on H3 HA-specific mucosal IgA response. Univariable logistic regression using n = 228 children. Excludes 4/244 participants with missing IgA data and 12/244 with shedding seen only at day 7. Univariable analysis with T-cell independent variables done with n = 206 children (excludes a further 22 children with missing T-cell data). Shedding coded as one variable with three levels (no shedding, day 2 only shedding, day 2 and day 7 shedding). No interactions observed between variables included in the multivariable analysis. OR = odds ratio, CI = confidence interval, HAI = haemagglutination inhibition

B/Vic seroconversion (2018)	Univariable			Multivariable		
	OR	95% CI	p value	OR	95% CI	p value
Age	0.95	0.90 - 1.00	0.06	0.96	0.89 - 5.16	0.26
Sex	1.96	0.81 - 5.03	0.15	1.75	0.58 - 5.56	0.32
Baseline seropositive (HAI)	**0.06**	**0.01 - 0.21**	**0.0002**	**0.13**	**0.02 - 0.57**	**0.02**
Day 2 shedding	**10.07**	**1.95 - 184.9**	**0.03**	-	-	-
Day 7 shedding	**23.31**	**6.35 - 151.3**	**<0.0001**	**10.47**	**2.35 - 75.9**	**0.006**
B/Vic HA CD4+IFNg-γ+ response	1.94	0.80 - 4.70	0.139	-	-	-
B/Vic HA CD4+IL2+ response	**3.99**	**1.63 - 10.38**	**0.003**	1.17	0.35 - 3.84	0.793

Table S12. Impact of B/Vic shedding on B/Vic seroconversion. Univariable and multivariable logistic regression using n = 109 children. Only participants from 2018 used as T-cell data were only generated to influenza B antigens in 2018. Excludes 17/126 participants with missing T-cell data. Day 2 and day 7 shedding variables included separately as combining into one variable (no shedding, day 2 only, day 2 and day 7) resulted in zero cell values (no seroconverters among those with no shedding). Only day 7 shedding taken forward to multivariable analysis. B/Vic Haemagglutinin (HA) CD4+IL-2+ response taken forward to multivariable analysis due to overlap between this and the CD4+IFN-γ response. No interactions observed between variables included in the multivariable analysis. OR = odds ratio, CI = confidence interval, HAI = haemagglutination inhibition.

B/Vic HA CD4+IFN-γ and/or IL-2 response (2018)	Univariable			Multivariable		
	OR	95% CI	p value	OR	95% CI	p value
Age	0.99	0.96 - 1.04	0.89	1.02	0.97 - 1.08	0.35
Sex	1.67	0.78 - 3.63	0.19	1.57	0.67 - 3.75	0.30
Baseline seropositive (HAI)	**0.23**	**0.10 - 0.51**	**0.0004**	0.40	0.15 - 1.02	0.06
Day 2 shedding	**2.87**	**1.12 - 7.82**	**0.03**	-	-	-
Day 7 shedding	**5.39**	**2.41 - 12.6**	**<0.0001**	**3.65**	**1.43 - 9.6**	**0.007**

Table S13. Impact of B/Vic shedding on B/Vic HA-specific CD4+ T-cell response (IFN-γ and/or IL-2). Univariable and multivariable logistic regression using n = 109 children. Only participants from 2018 used as T-cell data were only generated to influenza B antigens in 2018. Excludes 17/126 participants with missing T-cell data. Day 2 and day 7 shedding variables included separately as combining into one variable (no shedding, day 2 only, day 2 and day 7) resulted in zero cell values. Only day 7 shedding taken forward to multivariable analysis. No interactions observed between variables included in the multivariable analysis. OR = odds ratio, CI = confidence interval, HAI = haemagglutination inhibition

B/Vic HA IgA response (2018)	Univariable			Multivariable		
	OR	95% CI	p value	OR	95% CI	p value
Age	0.97	0.93 - 1.02	0.309	0.99	0.94 - 1.04	0.804
Sex	1.50	0.69 - 3.35	0.311	1.42	0.58 - 3.57	0.430
Baseline seropositive (HAI)	**0.81**	**0.69 - 0.95**	**0.010**	0.90	0.74 - 1.09	0.249
Day 2 shedding	1.30	0.55 - 3.27	0.553	-	-	-
Day 7 shedding	**3.94**	**1.76 - 9.29**	**0.001**	2.66	0.92 – 7.97	0.073
B/Vic HA CD4+IFNg+ response	**2.42**	**1.03 - 5.71**	**0.041**	-	-	-
B/Vic HA CD4+IL2+ response	**2.41**	**1.05 - 5.66**	**0.039**	1.32	0.48 - 3.62	0.679

Table S14. Impact of B/Vic shedding on B/Vic HA-specific mucosal IgA response. Univariable and multivariable logistic regression using n = 108 children. Only participants from 2018 used as T-cell data were only generated to influenza B antigens in 2018. Excludes 17/126 participants with missing T-cell data and 1/126 with poor mucosal IgA sample quality. Day 2 and day 7 shedding variables included separately as combining into one variable (no shedding, day 2 only, day 2 and day 7) resulted in zero cell values. Only day 7 shedding taken forward to multivariable analysis. B/Vic Haemagglutinin (HA) CD4+IL-2+ response taken forward to multivariable analysis due to overlap between this and the CD4+IFN-γ response. No interactions observed between variables included in the multivariable analysis. OR = odds ratio, CI = confidence interval, HAI = haemagglutination inhibition

		pH1N seroconversion			pH1 HA T-cell response			pH1 HA IgA response		
		Univariable			Univariable			Univariable		
		OR	95% CI	p value	OR	95% CI	p value	OR	95% CI	p value
Age		**0.92**	**0.85 - 0.96**	**0.03**	**0.94**	**0.89 - 0.98**	**0.01**	0.96	0.89 - 1.02	0.207
Sex		2.33	0.91 - 6.5	0.09	1.31	0.58 - 3.00	0.51	1.67	0.65 - 4.56	0.294
Baseline seropositive (HAI)*		-	-	-	-	-	-	-	-	-
Shedding	Day 2	3.24	0.58 - 24.85	0.19	1.18	0.41 - 3.45	0.76	1.07	0.24 - 4.51	0.929
	Day 2 and Day 7	**8.27**	**2.17 - 54.49**	**0.007**	**4.75**	**1.74 - 13.9**	**0.003**	1.77	0.60 - 6.04	0.323
pH1 HA CD4+IFNg+ response		**4.03**	**1.47 - 12.33**	**0.009**	-	-	-	0.61	0.19 - 1/74	0.358
pH1 HA CD4+IL2+ response		**5.31**	**1.78 - 19.7**	**0.005**	-	-	-	1.75	0.60 - 5.48	0.314

Table S15. Impact of NY15 pH1N1 shedding on pH1N1-specific immune responses. Univariable logistic regression analyses using children immunised with 2017-18 LAIV (n = 126 in total, n = 109 in T-cell analyses, as excludes 17/126 with missing T-cell data and n = 106 in IgA analyses, as excludes 5/126 with missing pH1N1 IgA data). *Unable to use baseline haemagglutination inhibition (HAI) titre as a variable in regression due to zero cell values (e.g. No seroconverters in those that are seropositive at baseline). Multivariable analysis not undertaken as main purpose is to adjust for effect of baseline HAI titre on shedding. Shedding coded as one variable with three levels (no shedding, day 2 only shedding and day 2 and day 7 shedding). HA = haemagglutinin, IFN-γ = interferon-gamma, IL-2 = interleukin-2, OR = odds ratio, CI = confidence interval.

Percentage of participants with T-cell responses to LAIV

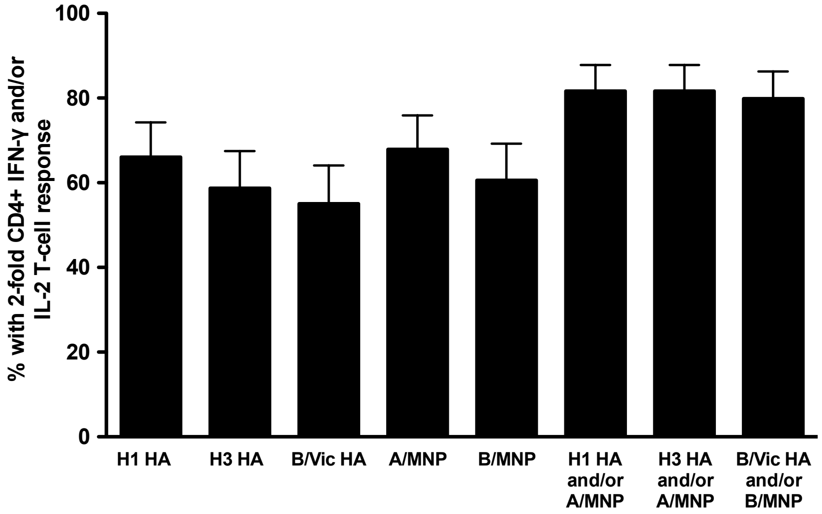

Figure S12. Percentage of children given the 2017-18 LAIV showing a 2-fold increase from baseline to day 21 post-LAIV, in interferon-gamma (IFN-γ) and/or interleukin-2 (IL-2) producing CD4+ T-cells. HA = haemagglutinin, MNP = matrix and nucleoprotein. Error bar represents upper 95% confidence interval.

References

1. Duvall MG, Precopio ML, Ambrozak DA, et al. Polyfunctional T cell responses are a hallmark of HIV-2 infection. European journal of immunology 2008; 38(2): 350-63.
2. de Silva TI, Peng Y, Leligdowicz A, et al. Correlates of T-cell-mediated viral control and phenotype of CD8(+) T cells in HIV-2, a naturally contained human retroviral infection. Blood 2013; 121(21): 4330-9.
3. Roederer M, Nozzi JL, Nason MC. SPICE: exploration and analysis of post-cytometric complex multivariate datasets. Cytometry Part A : the journal of the International Society for Analytical Cytology 2011; 79(2): 167-74.
4. National Institute of Public Health and the Environment. Annual report Surveillance of influenza and other respiratory infections in the Netherlands: winter 2017/2018. 2018. https://www.rivm.nl/bibliotheek/rapporten/2018-0049.pdf (accessed 18 Dec 2018).
5. Shcherbik S, Sergent SB, Davis WG, et al. Application of real time RT-PCR for the genetic homogeneity and stability tests of the seed candidates for live attenuated influenza vaccine production. Journal of virological methods 2014; 195: 18-25.
6. Stata Corp. Stata Statistical Software: Release 12. TX: StataCorp; 2011.
